# General model of nonradiative excitation energy migration on a spherical nanoparticle with attached chromophores

**DOI:** 10.1038/s41598-024-55193-4

**Published:** 2024-03-05

**Authors:** L. Kułak, A. Schlichtholz, P. Bojarski

**Affiliations:** 1 Faculty of Applied Physics and Mathematics, Gdańsk University of Technology, 80-233 Gdańsk, Poland; 2 Faculty of Mathematics, Physics and Informatics, University of Gdańsk, 80-308 Gdańsk, Poland

**Keywords:** Mathematics and computing, Nanoscience and technology, Physics

## Abstract

Theory of multistep excitation energy migration within the set of chemically identical chromophores distributed on the surface of a spherical nanoparticle is presented. The Green function solution to the master equation is expanded as a diagrammatic series. Topological reduction of the series leads to the expression for emission anisotropy decay. The solution obtained behaves very well over the whole time range and it remains accurate even for a high number of the attached chromophores. Emission anisotropy decay depends strongly not only on the number of fluorophores linked to the spherical nanoparticle but also on the ratio of critical radius to spherical nanoparticle radius, which may be crucial for optimal design of antenna-like fluorescent nanostructures. The results for mean squared excitation displacement are provided as well. Excellent quantitative agreement between the theoretical model and Monte–Carlo simulation results was found. The current model shows clear advantage over previously elaborated approach based on the Padé approximant.

## Introduction

Nowadays, various spherical nanoparticles are being intensively studied as nanomaterials. One example is core–shell spherical nanoparticles, which find wide applications in fields related to smart nanomaterials. These nanoparticles are used in targeted drug delivery, biosensing, bioimaging, DNA/RNA interaction, targeted gene delivery, photodynamic therapy, and plasmonically enhanced fluorescence^1–9^. Spherical nanostructures with multiple attached chromophores, achieved through covalent bonds, electrostatic interactions, or van der Waals forces, can serve as artificial light harvesting systems at the nanoscale. In these systems, excitation can repeatedly jump among the chromophores before being trapped in the reaction center.

Of particular interest is the scenario where multiple chemically identical fluorophores are present on the surface of the nanoparticle’s shell. In this case, these fluorophores can exchange energy through a long-range dipole–dipole mechanism after the initial excitation. This implies that the excitation energy can “jump” several times from one site to another, eventually reaching sites that are distant from the initially excited fluorophore. The path followed by the initial excitation among these sites before deexcitation can be complex. The main challenge lies in deriving an analytical expression for the fluorescence decay of the initially excited fluorophores, denoted as ${G}^{SD}\left(t\right)$, which is closely linked to the decay of emission anisotropy. This is because only the fluorophores that are initially excited contribute to the emission anisotropy^10–12^. However, finding a solution to this problem is difficult due to the immense number of possible excitation paths, which necessitates the use of approximations. The accuracy of the obtained expression heavily relies on the validity of these approximations.

Previous theories regarding the transport of electronic excitation between molecules in a solution have primarily focused on systems with infinite volume $V$ and an infinite number of particles $N$, which is known as the thermodynamic limit, ($\underset{N\to \infty , V\to \infty }{{\text{lim}}}\frac{N}{V}=const$). However, there are numerous experimental systems in which the volume or the number of chromophores is too small to apply the theory based on infinite volume solutions. Such cases frequently occur in the nanoscale regime and encompass situations such as chromophores attached to insulated polymer coils, photosynthetic antenna complexes, hybrid porous nanoparticles, nanolayers, and core–shell systems incorporating fluorophores, among others^13–17^.

An approximate theory has been developed to address the problem of excited state energy transport for chromophores confined to a microscopic-sized spherical finite volume^14^. In this theory, a truncated expansion in powers of the chromophore density is employed as an approximation for a one-component system. However, molecules located near the edge of the finite volume exhibit different neighbor distributions compared to those located inside the volume. This poses a challenge for the direct application of the diagrammatic technique since the finite volume breaks the translational invariance of the Green function, a property crucial for the topological reduction that allows for the inclusion of high-order terms in the chromophore density expansion.

Nevertheless, in the case of chromophores randomly distributed on the surface of a spherical nanoparticle, the appropriate Green function exhibits translational invariance. It is important to note that the diagrammatic method is highly flexible and can potentially be applied to any system where the molecule distribution is uniform and the system dynamics is described by a master equation (Chapman–Kolmogorov equation). This method provides an excellent framework for analyzing and understanding stochastic processes, such as multistep charge or energy transport in natural and artificial photosynthetic systems, dependencies of angular momentum on pressure and temperature, or the study of excitonic Rabi rotation in driven quantum dots.

Previously, the excited state transport on a spherical nanoparticle was treated using a simple Padé approximant to the density expansion of the system’s Green function^18^. However, this approximant only yields acceptable results for weak energy migration, which is applicable to relatively large nanoparticles with a small to moderate surface density of fluorophores.

In this work, the proposed three-body diagrammatic approach demonstrates excellent predictive capabilities regardless of the strength of energy migration. A comparison of the new results with the previous ones and Monte–Carlo simulations will be presented.

## Theory

### Description of the model

The problem of excited state transport in a one-component infinite disordered system in the thermodynamic limit, has been conveniently formulated by Haan and Zwanzig^19^. Gochanour, Andersen, and Fayer^20^ further extended this treatment by employing the diagrammatic technique to approximate the system’s Green function, which enables the calculation of various transport properties. However, when analyzing the chromophore system within a finite volume, the theoretical complexity of the excitation transport problem increases. In an infinite volume system, the ensemble-averaged Green function solution to the master equation is independent of the initial excitation position. This property allows for highly accurate approximations using nonperturbative techniques^21^. On the other hand, when chromophores are randomly distributed on the surface of a finite sphere, such as a core–shell nanoparticle, the local distribution of chromophores differs significantly from that in an infinite volume system. Additionally, the finite volume theory needs to account for the finite number of particles present in the system. Therefore, addressing the excited state transport problem within a finite volume requires a theoretical framework capable of handling these specific challenges. To establish notation and clearly define the problem, we will follow the initial steps outlined in our previous work^18^. We consider a system consisting of $N$ chemically linked chromophores (referred to as donors) randomly distributed on the surface of a spherical nanoparticle with radius $R$ and surface area $S$. Let us denote the donor molecules from $1$ to $N$. The individual configurations of the chromophores are described by the vector $\mathfrak{R}=\left({{\varvec{r}}}_{1},{{\varvec{r}}}_{2},\dots ,{{\varvec{r}}}_{N}\right),$ where ${{\varvec{r}}}_{i}$ represents the position of the *i*th donor molecule. Each of the excited donor molecules, denoted as ${D}^{*}$, can undergo deactivation through various physical processes, including fluorescence emission, non-radiative transitions, and non-radiative energy transfer from ${D}^{*}$ to another donor molecule $D$. The dynamics of excitation transfer in this core–shell system can be described by *master equation*

1 $$\frac{d{P}_{{x}_{i}{x}_{j}}^{\prime}\left(t\right)}{dt}=-\frac{1}{{\tau }_{0D}}{P}_{{x}_{i}{x}_{j}}^{\prime}\left(t\right)+\sum_{k=1,k\ne i}^{N}{w}_{{x}_{i}{x}_{k}}^{DD}{P}_{{x}_{k}{x}_{j}}^{\prime}\left(t\right)-\sum_{k=1,k\ne i}^{N}{w}_{{x}_{k}{x}_{i}}^{DD}{P}_{{x}_{i}{x}_{j}}^{\prime}\left(t\right), \quad 1 \le i\le N$$

In the master equation, ${P}_{{x}_{i}{x}_{j}}(t)$ represents the conditional probability density of finding the excitation on molecule ${x}_{i}$ at time $t$ given that molecule ${x}_{j}$ was initially excited at time $t = 0$. The transfer rate ${w}_{{x}_{i}{x}_{j}}^{DD}$ corresponds to the distance-dependent excitation transfer rate, which denotes the probability of transition per unit time from donor molecule $j$ to donor molecule $i$. It is important to note that ${w}_{{x}_{i}{x}_{i}}^{DD}$ is defined as zero ${(w}_{{x}_{i}{x}_{i}}^{DD}\equiv 0)$ to exclude self-transitions. The initial condition for the master equation is given by ${P}_{{x}_{i}{x}_{j}}^{\prime}\left(0\right)={\delta }_{ij}$, where ${\delta }_{ij}$ represents the Kronecker delta. By substituting ${P}_{{x}_{i}{x}_{j}}^{\prime}\left(t\right)={P}_{{x}_{i}{x}_{j}}\left(t\right) exp\left(-\frac{t}{{\tau }_{0D}}\right)$, where ${\tau }_{0D}$ represents the lifetime of a donor molecule in the absence of other molecules, we eliminate natural decay from the considerations. Then the *master equation* written in the matrix form is

2 $$\frac{d{\varvec{P}}\left(\mathfrak{R},t\right)}{dt}={\varvec{W}}\circ {\varvec{P}}\left(\mathfrak{R},t\right)$$

where

3 $${{\varvec{W}}}_{jk}={w}_{{x}_{j}{x}_{k}}^{DD}-{\delta }_{jk}\sum_{i=1}^{N}{w}_{{x}_{i}{x}_{k}}^{DD}, j\le N, k\le N$$

The searched Green function expressed by elements of matrix ${\varvec{W}}$ is of the form^20^

4 $$\mathcal{G}\text{(}{\varvec{r}}\text{, }{\varvec{r}}^{\prime}\text{, }\text{t }\text{)}=\frac{{\text{S}}}{N}\sum_{j=1}^{N}\sum_{k=1}^{N}{\langle \delta ({{\varvec{r}}}_{\text{j}}-{\varvec{r}})exp{(t {\varvec{W}})}_{jk}\delta ({{\varvec{r}}}_{\text{k}}-{\varvec{r}}^{\prime})\rangle }_{\mathfrak{R}}$$

where the bracket ${\langle \dots \rangle }_{\mathfrak{R}}$ signifies the ensemble average over the donor distribution $\mathfrak{R}.$

The Green function mentioned above represents the conditional probability density of finding an excitation at a point with coordinate ${\varvec{r}}$ at time $t$, given that the donor was initially excited at a point with coordinate ${{\varvec{r}}}^{\boldsymbol{^{\prime}}}$. The components in the double sum in Eq. (4) can be classified into two categories: diagonal terms where $j = k$, denoted as ${\mathcal{G}}^{SD}\text{(}{\varvec{r}}\text{, }{\varvec{r}}^{\prime}\text{, }\text{t }\text{)}$, and non-diagonal terms where $j$ $\ne$ $k$, denoted as ${\mathcal{G}}^{DD}\text{(}{\varvec{r}}\text{, }{\varvec{r}}^{\prime}\text{, }\text{t }\text{)}$.

The components of the Green function can be expanded into diagrammatic series. To do this it is convenient to work with the Fourier–Laplace transforms of ${\mathcal{G}}^{SD}\text{(}{\varvec{r}}\text{, }{\varvec{r}}^{\prime}\text{, }\text{t }\text{)}$ and ${\mathcal{G}}^{DD}\text{(}{\varvec{r}}\text{, }{\varvec{r}}^{\prime}\text{, }\text{t }\text{)}$, which are given by

5 $${\widehat{G}}^{SD}\left(\epsilon \right)={\langle {\left[{\left(\epsilon {\mathbb{I}}-{\varvec{W}}\right)}^{-1}\right]}_{11}\rangle }_{\mathfrak{R}}$$

6 $${\widehat{G}}^{DD}\left({\varvec{k}},\epsilon \right){=\left(N-1\right)\langle {exp\left(i{\varvec{k}}{{\varvec{r}}}_{12}\right)\left[{\left(\epsilon {\mathbb{I}}-{\varvec{W}}\right)}^{-1}\right]}_{21}\rangle }_{\mathfrak{R}}$$

where ${\mathbb{I}}$ is the identity matrix. The Green functions ${\widehat{G}}^{DD}\left({\varvec{k}},\epsilon \right)$ and ${\widehat{G}}^{SD}\left(\epsilon \right)$ are not independent. In order to maintain physical consistency and preserve probability, it is necessary for a physically acceptable solution that $\underset{{\varvec{k}}\to 0}{\mathit{lim}}\widehat{G}\left({\varvec{k}},\epsilon \right)=\frac{1}{\epsilon } .$ This requirement leads us to derive the following equation, which connects these Green functions and is commonly known as the self-consistent equation

7 $$\epsilon \left[{\widehat{G}}^{SD}\left(\epsilon \right)+{\widehat{G}}^{DD}\left({\varvec{k}}=0,\epsilon \right)\right]=1$$

The diagrammatic series for both Green functions, ${\widehat{G}}^{DD}\left({\varvec{k}},\epsilon \right)$ and ${\widehat{G}}^{SD}\left(\epsilon \right)$, can be obtained by expanding the matrix ${\left(\epsilon {\mathbb{I}}-{\varvec{W}}\right)}^{-1}$ in powers of $\epsilon$ and ${\varvec{W}}$ (for more detailed information, refer to the Supporting Information A)^20^. In the equation for ${\widehat{G}}^{SD}\left(\epsilon \right)$, each $n-th$ term is composed of an $n$-fold product of ${{\varvec{W}}}_{{1i}_{1}}{{\varvec{W}}}_{{i}_{1}{i}_{2}}...{{\varvec{W}}}_{{i}_{n}1}$. Examining the definition of the matrix ${\varvec{W}}$ reveals that individual elements, ${{\varvec{W}}}_{ij}$, can be a single term when $i = j$ or a sum of $N$ terms when $i\neq j$. Consequently, each $n$-fold product of ${{\varvec{W}}}_{ij}$, where $m$ of them are diagonal $(i = j)$, generates ${N}^{m}$ products of $n$-element transfer rates ${w}_{{x}_{i}{x}_{j}}^{DD}.$

To obtain the appropriate Green function, a specialized computational technique is necessary when summing all these products as $n$ approaches infinity. The diagrammatic method is the appropriate approach for this purpose. To establish the notation and provide a precise definition of the diagrammatic method, we follow the initial steps of the GAF theory^20,22^, which we have adapted for finite-volume systems. For this purpose, we define a diagrammatic representation, or graph, to depict the transfer rates ${w}_{{x}_{i}{x}_{j}}^{DD}$. The vertices of the graph are marked with donor numbers, while directed arrows represent each transfer rate ${w}_{{x}_{i}{x}_{j}}^{DD}$. In this representation, arrows ${w}_{{x}_{i}{x}_{j}}^{DD}$ representing the transfer rate from donor molecule $j$ to donor molecule $i$ are drawn as continuous lines, whereas arrows corresponding to (${-w}_{{x}_{i}{x}_{j}}^{DD}$) are initially drawn as solid lines and then changed to dashed lines. The sign ($+$ or $-$) assigned to the arrow ${w}_{{x}_{i}{x}_{j}}^{DD}$ indicates an increase or decrease in the transfer rate ${w}_{{x}_{i}{x}_{j}}^{DD}$ during a given transition. A path in the graph that begins and ends at the same vertex is referred to as a loop. It can be observed that the multigraphs corresponding to the products ${w}_{{x}_{2}{x}_{1}}^{DD}{w}_{{x}_{3}{x}_{2}\dots }^{DD}{w}_{{x}_{{i}_{n}}{x}_{{i}_{n-1}}}^{DD}$, form paths in the graph. We assign the value ${\epsilon }^{-1}$ to the vertices corresponding to the donors. To each multigraph, we assign a numerical value by taking the ensemble average of the product formed by a factor of ${\epsilon }^{-1}$ for each donor vertex, a factor of ${w}_{{x}_{i}{x}_{j}}^{DD}$ for each solid arrow, and a factor of $(-1)$ for each dashed arrow. As a result, the Green function ${\widehat{G}}^{SD}\left(\epsilon \right)$ can be represented by the following diagrammatic series: ${\widehat{G}}^{SD}\left(\epsilon \right)$ = ${\epsilon }^{-1}+$ sum of all multigraphs consisting of loops starting from vertex number $1$. Similarly, the Green function ${\widehat{G}}^{DD}\left({\varvec{k}},\epsilon \right)$ can be represented by the diagrammatic series: ${\widehat{G}}^{DD}\left({\varvec{k}},\epsilon \right)$ = sum of all different (topologically different) multigraphs consisting of paths starting from vertex number $1$ and ending at vertex number $2$. The complexity of the diagrammatic series can be reduced through a topological reduction procedure. This involves examining the topological structures of the diagrammatic series for ${\widehat{G}}^{DD}\left({\varvec{k}},\epsilon \right)$ to identify a smaller set of diagrams from which all other diagrams can be generated. One observation is that some diagrams in the series contain loops. By removing the maximal subgraph that forms a loop within a given diagram and replacing its beginning and end with a single vertex, the multigraphs with loops can be renormalized. The resulting transformed multigraph represents the class of all multigraphs with the same set of donors connected by arrows and loops. The value of the transformed multigraph (without loops) is calculated by substituting the factors ${\epsilon }^{-1}$ associated with the vertex with ${\widehat{G}}^{SD}\left(\epsilon \right)$. Another topological property of the multigraphs in the series, representing the Green function ${\widehat{G}}^{DD}\left({\varvec{k}},\epsilon \right)$, is the presence of nodes. A node is defined as a vertex in the multigraph that separates it into two disjoint subgraphs. The value of any multigraph (without loops) containing nodes is obtained by multiplying the values of the individual subgraphs into which it is divided. Donor nodes are assigned a factor of $1/{\widehat{G}}^{SD}\left(\epsilon \right)$. Finally, the diagrammatic series can be further renormalized through the loops and node removal procedure. By introducing the diagrammatic series ${\widehat{\Sigma }}^{DD}\left({\varvec{k}},\epsilon ,{\widehat{G}}^{SD}\left(\epsilon \right)\right)$ which is the sum of all multigraphs with no loops and no nodes starting at any fixed donor (e.g. $1$) and ending at any fixed donor different from the initial donor (e.g. $2$), we obtain^20,22^

8 $${\widehat{G}}^{DD}\left({\varvec{k}},\epsilon \right)=\frac{{\widehat{\Sigma }}^{DD}}{ 1-{\widehat{\Sigma }}^{DD}/{\widehat{G}}^{SD}}$$

In the self-consistent approximation^20,22^, we treat the function ${\widehat{G}}^{SD}\left(\epsilon \right)$ as an unknown function. We partially sum the diagrammatic series for ${\widehat{\Sigma }}^{DD}\left({\varvec{k}},\epsilon ,{\widehat{G}}^{SD}\left(\epsilon \right)\right)$ to obtain approximations to the function ${\widehat{G}}^{DD}\left({\varvec{k}},\epsilon \right)$ in terms of the unknown ${\widehat{G}}^{SD}\left(\epsilon \right)$. Substituting this approximation into Eq. (7), the resulting equation involves only known quantities and the function ${\widehat{G}}^{SD}\left(\epsilon \right)$, allowing us to solve for ${\widehat{G}}^{SD}\left(\epsilon \right)$. In the considered case, it leads to a third-degree polynomial equation, which always has one real root. This root can be calculated using Cardano’s formulas.

We can provide a physical interpretation of the function ${\widehat{\Sigma }}^{DD}\left({\varvec{k}},\epsilon ,{\widehat{G}}^{SD}\left(\epsilon \right)\right)$. It represents the Fourier–Laplace transform of the transition probabilities per unit time (transfer rates) for energy ”jumps” that are not correlated with past and subsequent jumps. In an ensemble of molecules, energy excitation jumps occur until it reaches a node, and once it leaves that node, it never returns to the same assembly. Therefore, ${\widehat{\Sigma }}^{DD}\left({\varvec{k}},\epsilon ,{\widehat{G}}^{SD}\left(\epsilon \right)\right)$ is associated with the migration of excitation energy within the set of donors. It is important to note that ${\widehat{\Sigma }}^{DD}\left({\varvec{k}},\epsilon ,{\widehat{G}}^{SD}\left(\epsilon \right)\right)$ does not represent an energy jump in a single step. This is because the series is already renormalized, meaning that the vertex is assigned ${\widehat{G}}^{SD}\left(\epsilon \right)$, which contains loops. These loops, in theory, can even consist of an infinite number of energy jumps. Furthermore, based on the definition of a loop, there is no limit to the number of jumps that can occur until a node is reached. We can establish a connection between the theoretical results and experimentally measured physical quantities. Firstly, the decay of primarily excited donor fluorescence can be determined using the formula $I\left(t\right)={I}_{0}\mathit{exp}\left(-\frac{t}{{\tau }_{0D}}\right) {G}^{SD}\left(t\right)$, where ${\tau }_{0D}$ represents the donor lifetime in the absence of other molecules. To obtain the time-domain expression for the function ${G}^{SD}\left(t\right)$ we need to invert the Laplace transform of ${\widehat{G}}^{SD}\left(\epsilon \right)$, i.e. ${G}^{SD}\left(t\right)={\mathcal{L}}_{\epsilon }^{-1}\left({\widehat{G}}^{SD}\left(\epsilon \right)\right)$. Next, the parallel and perpendicular components of the decay of donor fluorescence can be obtained from the following equations^12^

9 $${I}_{\parallel }\left(t\right)={e}^{-t/{\tau }_{0D}}\left(1+\frac{4}{5} {G}^{SD}\left(t\right)\right)$$

10 $${I}_{\perp }\left(t\right)={e}^{-t/{\tau }_{0D}}\left(1- \frac{2}{5}{G}^{SD}\left(t\right)\right)$$

Finally, we can calculate the experimentally measurable decay of the emission anisotropy using the following formula

11 $$r\left(t\right)=\frac{{I}_{\parallel }\left(t\right)-{I}_{\perp }\left(t\right)}{{I}_{\parallel }\left(t\right)+2 {I}_{\perp }\left(t\right)}$$

By evaluating this equation, we can obtain the time-resolved decay of the emission anisotropy, which provides valuable information about the rotational dynamics and molecular interactions of the fluorophores.

## Two- and three-body approximation: general equations

In order to obtain the time decays and stationary values of the observables that characterize the studied system, it is sufficient to know the values of the diagrammatic series ${\widehat{\Sigma }}^{DD}\left({\varvec{k}},\epsilon ,{\widehat{G}}^{SD}\left(\epsilon \right)\right)$. However, this series cannot be exactly summed up, necessitating the use of approximate methods. One such method is the two-body approximation, which involves restricting calculations of the function ${\widehat{\Sigma }}^{DD}\left({\varvec{k}},\epsilon ,{\widehat{G}}^{SD}\left(\epsilon \right)\right)$ to only two-body multigraphs. Similarly, in the three-body approximation, we consider both two-body and three-body multigraphs. Subsequently, we employ the self-consistent method for the fundamental observables. This method involves treating the function ${\widehat{G}}^{SD}\left(\epsilon \right)$ as the variable to be determined after obtaining an approximate expression for ${\widehat{\Sigma }}^{DD}\left({\varvec{k}},\epsilon ,{\widehat{G}}^{SD}\left(\epsilon \right)\right)$. This is achieved by substituting Green's functions ${\widehat{G}}^{DD}\left({\varvec{k}},\epsilon \right)$, which depend on ${\widehat{\Sigma }}^{DD}\left({\varvec{k}},\epsilon ,{\widehat{G}}^{SD}\left(\epsilon \right)\right)$, into the self-consistent Eq. (7). By following this procedure, the accuracy of the calculations is significantly enhanced, and it is simpler compared to other methods that involve approximating both the functions ${\widehat{G}}^{DD}\left({\varvec{k}},\epsilon \right)$ and ${\widehat{G}}^{SD}\left(\epsilon \right)$ using appropriate expansions into diagrammatic series.

In the two-body approximation adapted for finite-volume systems, the function ${\widehat{\Sigma }}^{DD}\left({\varvec{k}},\epsilon ,{\widehat{G}}^{SD}\left(\epsilon \right)\right)$ is represented as the infinite sum of a series of two-body multigraphs that do not contain loops or nodes^20,22^

12 $${\widehat{\Sigma }}_{2}^{DD}\left(0,\epsilon ,{\widehat{G}}^{SD}\left(\epsilon \right)\right)=\frac{N-1}{S} \int d{{\varvec{r}}}_{12}\frac{{\left({\widehat{G}}^{SD}\right)}^{2}{w}_{{x}_{2}{x}_{1}}^{DD}}{1+2 {\widehat{G}}^{SD}{w}_{{x}_{2}{x}_{1}}^{DD}}$$

In the three-body approximation adapted for finite-volume systems, it is necessary to sum all three-body multigraphs ${\widehat{{\text{A}}}}_{3}^{DDD}$ and then subtract the three-body multigraphs with loops ${\widehat{{\text{L}}}}_{3}^{DDD}$ and the three-body multigraphs with nodes ${\widehat{{\text{N}}}}_{3}^{DDD}$. This process ensures that the resulting calculation includes the contributions from all three-body interactions while excluding the effects of loops and nodes.

13 $${\widehat{\Sigma }}_{3}^{DDD}\left({\varvec{k}},\epsilon ,{\widehat{G}}^{SD}\left(\epsilon \right)\right)={\widehat{{\text{A}}}}_{3}^{DDD}\left({\varvec{k}},\epsilon ,{\widehat{G}}^{SD}\left(\epsilon \right)\right)-{\widehat{{\text{L}}}}_{3}^{DDD}\left({\varvec{k}},\epsilon ,{\widehat{G}}^{SD}\left(\epsilon \right)\right)-{\widehat{{\text{N}}}}_{3}^{DDD}\left({\varvec{k}},\epsilon ,{\widehat{G}}^{SD}\left(\epsilon \right)\right)$$

The individual multigraph series are as follows.

– Sum of all three-body multigraphs

14 $${\widehat{{\text{A}}}}_{3}^{DDD}\left({\varvec{k}},\epsilon \right)=\frac{\left(N-1\right)\left(N-2\right)}{{S}^{2}}\int d{{\varvec{r}}}_{12}\mathit{exp}\left(i{\varvec{k}}{{\varvec{r}}}_{12}\right)\int d{{\varvec{r}}}_{13}\left\{A\left({{\varvec{r}}}_{12},{{\varvec{r}}}_{13},{\widehat{G}}^{SD}\right)-\frac{{\left({\widehat{G}}^{SD}\right)}^{2}{w}_{{x}_{2}{x}_{1}}^{DD}}{1+2{\widehat{G}}^{SD}{w}_{{x}_{2}{x}_{1}}^{DD}}\right\}$$

The function under the integral $A\left({{\varvec{r}}}_{12},{{\varvec{r}}}_{13},\epsilon \right)={A}_{L}\left({{\varvec{r}}}_{12},{{\varvec{r}}}_{13},\epsilon \right)/{A}_{M}\left({{\varvec{r}}}_{12},{{\varvec{r}}}_{13},\epsilon \right)$ is

15 $${A}_{L}\left({{\varvec{r}}}_{12},{{\varvec{r}}}_{13},{\widehat{G}}^{SD}\right)={\left({\widehat{G}}^{SD}\right)}^{2}\left[{w}_{{x}_{2}{x}_{1}}^{DD}+{\widehat{G}}^{SD}\left({w}_{{x}_{2}{x}_{1}}^{DD}{w}_{{x}_{3}{x}_{1}}^{DD}+{w}_{{x}_{2}{x}_{1}}^{DD}{w}_{{x}_{3}{x}_{2}}^{DD}+{w}_{{x}_{3}{x}_{1}}^{DD}{w}_{{x}_{3}{x}_{2}}^{DD}\right)\right]$$

16 $${A}_{M}\left({{\varvec{r}}}_{12},{{\varvec{r}}}_{13},{\widehat{G}}^{SD}\right)=1+{2 \widehat{G}}^{SD}\left({w}_{{x}_{2}{x}_{1}}^{DD}+{w}_{{x}_{3}{x}_{1}}^{DD}+{w}_{{x}_{3}{x}_{2}}^{DD}\right){+3 \left({\widehat{G}}^{SD}\right)}^{2}\left({w}_{{x}_{2}{x}_{1}}^{DD}{w}_{{x}_{3}{x}_{2}}^{DD}+{w}_{{x}_{2}{x}_{1}}^{DD}{w}_{{x}_{3}{x}_{1}}^{DD}+{w}_{{x}_{3}{x}_{1}}^{DD}{w}_{{x}_{3}{x}_{2}}^{DD}\right)$$

– Sum of all three-body multigraphs with loops

17 $${\widehat{{\text{L}}}}_{3}^{DDD}\left({\varvec{k}},\epsilon ,{\widehat{G}}^{SD}\left(\epsilon \right)\right)=2\frac{\left(N-1\right)\left(N-2\right)}{{S}^{2}}{B}_{2}^{SD}\left(\epsilon \right)\int d{{\varvec{r}}}_{12}exp\left(i{\varvec{k}}{{\varvec{r}}}_{12}\right)\frac{{\widehat{G}}^{SD}{w}_{{x}_{2}{x}_{1}}^{DD}\left(1+{\widehat{G}}^{SD}{w}_{{x}_{2}{x}_{1}}^{DD}\right)}{{\left(1+{2 \widehat{G}}^{SD}{w}_{{x}_{2}{x}_{1}}^{DD}\right)}^{2}}$$

where

18 $${B}_{2}^{SD}\left(\epsilon \right)=-\int d{{\varvec{r}}}_{12}\frac{{ \left({\widehat{G}}^{SD}\right)}^{2}{w}_{{x}_{2}{x}_{1}}^{DD}}{\left(1+{2 {\widehat{G}}^{SD}w}_{{x}_{2}{x}_{1}}^{DD}\right)}$$

– Sum of all three-body multigraphs with nodes

19 $${\widehat{{\text{N}}}}_{3}^{DDD}\left({\varvec{k}},\epsilon ,{\widehat{G}}^{SD}\left(\epsilon \right)\right)=\frac{{\left(N-1\right)}^{2}}{{S}^{2}}{\left({\widehat{G}}^{SD}\right)}^{3}\int d{{\varvec{r}}}_{12}\int d{{\varvec{r}}}_{13} exp\left(i{\varvec{k}}{{\varvec{r}}}_{13}\right)N\left({{\varvec{r}}}_{12},{{\varvec{r}}}_{13},{\widehat{G}}^{SD}\right)$$

where

20 $$N\left({{\varvec{r}}}_{12},{{\varvec{r}}}_{13},{\widehat{G}}^{SD}\right)=\frac{{w}_{{x}_{2}{x}_{1}}^{DD}}{1+2 {\widehat{G}}^{SD}{w}_{{x}_{2}{x}_{1}}^{DD}}\cdot \frac{{w}_{{x}_{3}{x}_{2}}^{DD}}{1+2 {\widehat{G}}^{SD}{w}_{{x}_{3}{x}_{2}}^{DD}}$$

The values of the above diagrammatic series can be calculated numerically by evaluating the corresponding quadruple integrals for the finite-volume system. The computation results are presented in Supporting Information B.

## Results and discussion

### Numerical calculations

In the following discussion, we will consider dipole–dipole mechanism of energy migration within the set of donors. For this physical process, the transfer rate is given by ${w}_{{x}_{i}{x}_{j}}^{DD}=\frac{1}{{\tau }_{0D}}{\left(\frac{{R}_{0}^{DD}}{{r}_{ij}}\right)}^{6}$^23^. In this formula, ${\tau }_{0D}$ is a donor lifetime in the absence of other molecules, ${R}_{0}^{DD}$ is the critical radius (distance) for energy transfer.

Then, for ${\varvec{k}}=0$, after calculating the appropriate integrals in spherical coordinates (see Supporting Information B), we obtain the result for the two-body approximation

21 $${\widehat{\Sigma }}_{2}^{DD}\left(0,\epsilon ,{\widehat{G}}^{SD}\left(\epsilon \right)\right)\approx \left(N-1\right)\frac{{\widehat{G}}^{SD}}{36} \sqrt[3]{2} \sqrt{3} \pi \cdot {\left(\frac{{ R}_{0}^{DD}}{R}\right)}^{2}{\left(\frac{ {\widehat{G}}^{SD}}{{\tau }_{0D}}\right)}^\frac{1}{3}$$

In the three-body approximation we have

22 $${\widehat{\Sigma }}^{DD}\left({\varvec{k}},\epsilon ,{\widehat{G}}^{SD}\left(\epsilon \right)\right)={\widehat{\Sigma }}_{2}^{DD}\left({\varvec{k}},\epsilon ,{\widehat{G}}^{SD}\left(\epsilon \right)\right)+{\widehat{\Sigma }}_{3}^{DD}\left({\varvec{k}},\epsilon ,{\widehat{G}}^{SD}\left(\epsilon \right)\right)$$

The above function can be obtained by numerically calculating the appropriate quadruple integrals in Eq. (13) (see Supporting Information B), resulting in

23 $${\widehat{\Sigma }}^{DD}\left({\bf0},\epsilon , {\widehat{G}}^{SD}\left(\epsilon \right)\right)\approx \left(N-1\right)\frac{{\widehat{G}}^{SD}}{36} \sqrt[3]{2} \sqrt{3} \pi \cdot {\left(\frac{{ R}_{0}^{DD}}{R}\right)}^{2}{\left(\frac{{\widehat{G}}^{SD}}{{\tau }_{0D}}\right)}^\frac{1}{3} - 0.00954125 \left(N-1\right) \left(N-2\right) {\widehat{G}}^{SD}{\left(\frac{{ R}_{0}^{DD}}{R}\right)}^{4}{\left(\frac{{\widehat{G}}^{SD}}{{\tau }_{0D}}\right)}^\frac{2}{3}$$

The self-consistent Eq. (7) expressed by the diagrammatic series ${\widehat{\Sigma }}^{DD}\left({\bf0},\epsilon ,{\widehat{G}}^{SD}\left(\epsilon \right)\right)$ takes the form of

24 $${\widehat{G}}^{SD}\left(\epsilon \right)=\frac{ 1/\epsilon }{1+\frac{{\widehat{\Sigma }}^{DD}\left(0,\epsilon ,{\widehat{G}}^{SD}\right)}{\epsilon { \left({\widehat{G}}^{SD}\right)}^{2}}}$$

By inserting the expression for ${\widehat{\Sigma }}^{DD}\left({\bf0},\epsilon ,{\widehat{G}}^{SD}\left(\epsilon \right)\right)$ into Eq. (24), substituting ${x=\left( \frac{{\widehat{G}}^{SD}\left(\epsilon \right)}{{\tau }_{0D}}\right)}^\frac{1}{3}$ and introducing the parameter $\xi ={R}_{0}^{DD}/R$ we obtain a third-degree polynomial equation

25 $${x}^{3}- \left(N-1\right)\left(N-2\right) \frac{ 0.00954125 }{\epsilon {\tau }_{0D}} {\xi }^{4} {x}^{2}+\left(N-1\right)\frac{\sqrt[3]{2} \sqrt{3} \pi }{36 \epsilon {\tau }_{0D}} \cdot {\xi }^{2} x-\frac{1}{\epsilon {\tau }_{0D}}=0$$

This equation always has one real root, which can be found using Cardano's formula.

## Comparison with Monte Carlo simulation results

Below, sample graphs are shown comparing the results of Monte-Carlo simulations with the previous model based on the Padé approximant and a general diagrammatic model.

**Figure 1:**
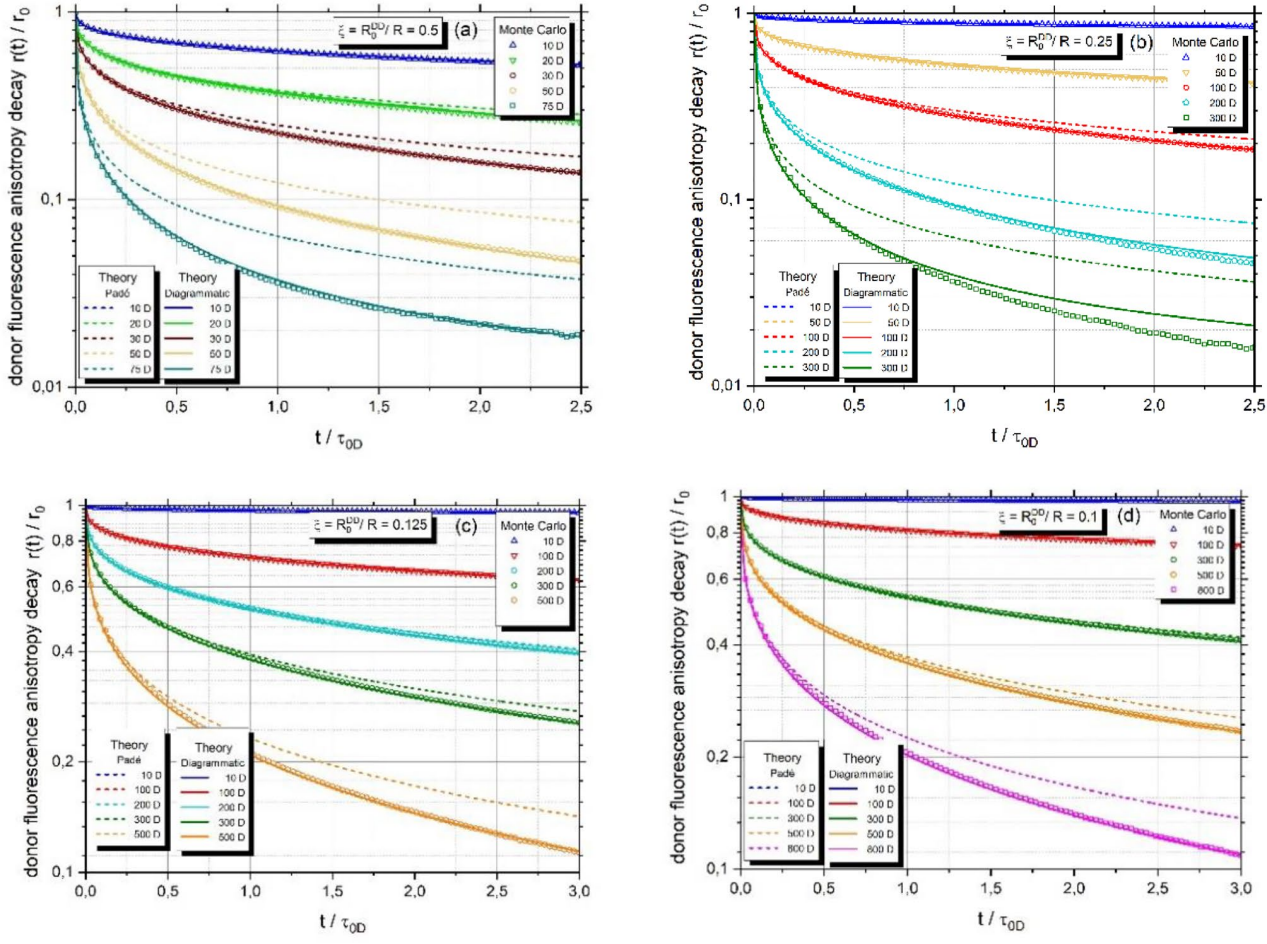
(**a**–**d**) Comparison of the Monte Carlo simulation results with Eq. (11) for a different number of donors attached to spherical nanoparticle and various values of the parameter $\xi ={R}_{0}^{DD}/R$. (**a**) $\xi =0.5,$ (**b**) $\xi =0.25$, (**c**) $\xi =0.125$ and (**d**) $\xi =0.1$. Dashed curves correspond to simplified model based on Padé approximant. Theoretical calculations of donor fluorescence anisotropy decay $r(t)/{r}_{0}$ in the three-body approximation obtained from Eq. (11) were made by inverting Laplace transform of ${\widehat{G}}^{SD}\left(\epsilon \right)$ in Eq. (25).

As shown in Fig. 1, the superiority of the diagrammatic method over the Padé approximant becomes clear at long times after excitation, as evidenced by the Monte-Carlo results which are in excellent agreement with the new model in any case. The advantage of new approach is especially evident for higher values of $\xi$ corresponding to smaller nanoparticles (Fig. 1a and b) and strong energy migration (high number of fluorophores). Comparable outcomes predicted by both models are limited to the case of weak energy migration especially well illustrated in Fig. 1c and d (larger nanoparticles with relatively low number of fluorophores attached to the surface).

From the mathematical point of view Padé approximant considers only three-body diagrams of the type ${\widehat{{\text{A}}}}_{3}^{DDD}$, supplemented by the substitution of ${\widehat{G}}^{SD}\left(\epsilon \right)\to {\epsilon }^{-1}$. Consequently, the Padé approximant’s capability to encompass a broader range of excitation energy transport pathways is significantly limited. Conversely, when the diagrammatic method incorporates loops ${\widehat{{\text{L}}}}_{3}^{DDD}$ and nodes ${\widehat{{\text{N}}}}_{3}^{DDD}$ into the calculations, the obtained results align well with those of the Monte–Carlo method.

**Figure 2:**
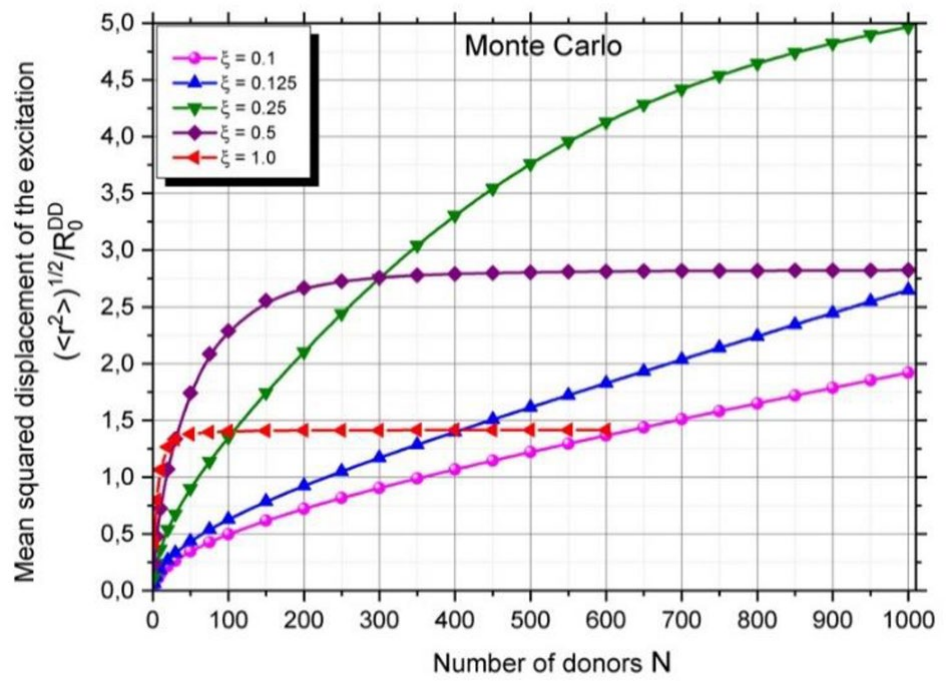
Monte Carlo simulation results of the relative mean squared displacement of the excitation ${\langle {r}^{2}\rangle }^{1/2}/{R}_{0}^{DD}$. The results of Monte Carlo simulation as a function of the number of donors attached to a spherical nanoparticle for different values of the parameter $\xi =\frac{{R}_{0}^{DD}}{R} .$

It is intriguing to explore the extent to which energy migration on a spherical nanoparticle can be considered a local effect. To address this Fig. 2 illustrates the results of the Monte–Carlo simulation, depicting the relative mean squared displacement of the excitation, ${\langle {r}^{2}\rangle }^{1/2}/{R}_{0}^{DD}$, as a function of the number $N$ of attached donors, for various values of the parameter $\xi =\frac{{R}_{0}^{DD}}{R}$, where R is the nanoparticle radius.

The findings from the Monte–Carlo simulation, as depicted in Fig. 2, reveal that the relative mean squared displacement of the excitation, ${\langle {r}^{2}\rangle }^{1/2}/{R}_{0}^{DD}$, exhibits a nonlinear relationship with the number N of donor molecules attached to the spherical nanoparticles. For a very small nanoparticle ($\xi =\frac{{R}_{0}^{DD}}{R}=1)$ the mean squared displacement of the excitation grows very rapidly and reaches plateau value equal to about 1,5 times the radius of the nanoparticle. This indicates that the energy migration, even within a relatively small set of fluorophores (N = 50), is a nonlocal process, occurring across the entire surface of nanoparticle. As the size of nanoparticle increases, mean squared displacement grows less dynamically and for three lowest values of $\xi$ it does not reach plateau even for very a high number of fluorophores. For large nanoparticle $(\xi =0.1$) energy migration becomes localized. In this case the relative mean squared displacement is about 0.03 R for $N=50$ and it increases only to 0.2 R even for extremely high number of fluorophores N = 1000. This means that we should treat energy migration as a process occurring in the vicinity of primarily excited site (local process).

## Conclusions

In summary, the presented model of energy migration on a spherical nanoparticle with randomly distributed donors on its surface demonstrates strong sensitivity to the number of donors and the nanoparticle's radius. The Monte Carlo simulations validate the model across all simulated scenarios, providing excellent agreement in terms of relative emission anisotropy decay. Furthermore, the simulations validate the universality of the diagrammatic method used in this study. It consistently produces results that align remarkably well with the Monte Carlo simulations, regardless of the ratio of nanoparticle radius to critical distance or the number of fluorophores. In contrast, the three-body Padé approximant is limited in its effectiveness, delivering satisfactory results only for weak energy migration. Thus, the developed model is a valuable tool for estimating the mean number of chromophores bound to spherical nanoparticles and designing optimal nanoparticles in terms of radius and critical distance for energy migration (${R}_{0}^{DD}$). The results show that on large nanoparticles, excitation mostly occurs at sites close to the initially excited one, indicating a local process. This is in contrast to small nanoparticles where excitation occurs at sites distal from the primarily excited molecule.

Currently, we are working on specific extensions and applications of the primary model presented here. The model for energy migration on a spherical nanoparticle has potential for extensions, including energy transport models in specific nanostructures containing several chemically different fluorophores with broadband emission. Such systems may be suitable as sources of white light in nanoscale. Further modifications to the model may include core–shell nanoparticles with metallic cores designed to enhance energy transfer through plasmonic resonance, as well as aggregated core–shell nanosystems as a part of energy transfer-based plasmonic platforms for highly sensitive detection of biospecies.

### Supplementary Information

Supplementary Information.

## Data Availability

No new data were generated or analyzed in the presented research.
