# Supplementary material for: General model of nonradiative excitation energy migration on a spherical nanoparticle with attached chromophores
Source: Sci Rep. 2024 Mar 5;14:5479. doi: 10.1038/s41598-024-55193-4 (PMC11319653; doi:10.1038/s41598-024-55193-4)
Supplement: Supplementary file 1 — Supplementary Information. [file 41598_2024_55193_MOESM1_ESM.pdf]

SUPLEMENTARY MATERIAL

General Model of Nonradiative Excitation Energy Migration on a Spherical Nanoparticle with Attached Chromophores

L. Kułak[1] A. Schlichholz[2] and P. Bojarski[2,*]

[1]*Faculty of Applied Physics and Mathematics, Gdańsk University of Technology, 80-233 Gdańsk, Poland*

[2]*Faculty of Mathematics, Physics and Informatics, University of Gdańsk, 80-308 Gdańsk, Poland*

[*] corresponding Author: piotr.bojarski@ug.edu.pl (Piotr Bojarski)

Supporting Information A: DIAGRAMMATIC SERIES FOR GREEN'S FUNCTIONS

To solve problem of nonradiative excitation energy migration on a spherical nanoparticle we find the solution of the master equation using the so-called Green function method. The components of the searched Green function can be expanded in diagrammatic series. The Fourier-Laplace transforms of Green's functions $\mathcal{G}^{SD}(\boldsymbol{r}, \boldsymbol{r}', t)$ and $\mathcal{G}^{DD}(\boldsymbol{r}, \boldsymbol{r}', t)$, are given by

$$\hat{G}^{SD}(\epsilon) = \langle [(\mathbb{E} - \boldsymbol{W})^{-1}]_{11} \rangle_{\mathfrak{R}} \qquad (S1)$$

$$\hat{G}^{DD}(\boldsymbol{k}, \epsilon) = (N-1) \langle exp(i\boldsymbol{k}\boldsymbol{r}_{12}) [(\mathbb{E} - \boldsymbol{W})^{-1}]_{21} \rangle_{\mathfrak{R}} \qquad (S2)$$

where the bracket $\langle \ldots \rangle_{\mathfrak{R}}$ signifies the ensemble average over the donor distribution $\mathfrak{R}$ and we used substitution $\mathbb{E} = \epsilon\, \mathbb{I}$.

By iterating the operator identity

$$(\mathbb{E} - \boldsymbol{W})^{-1} = \mathbb{E}^{-\mathbf{1}} + \mathbb{E}^{-\mathbf{1}} \circ \boldsymbol{W} \circ (\mathbb{E} - \boldsymbol{W})^{-1} \qquad (S3)$$

we obtain

$$(\mathbb{E} - \boldsymbol{W})^{-1} = \mathbb{E}^{-\mathbf{1}} + \mathbb{E}^{-\mathbf{1}} \circ \boldsymbol{W} \circ \mathbb{E}^{-\mathbf{1}} + \mathbb{E}^{-\mathbf{1}} \circ \boldsymbol{W} \circ \mathbb{E}^{-\mathbf{1}} \circ \boldsymbol{W} \circ \mathbb{E}^{-\mathbf{1}} + \cdots \qquad (S4)$$

From Eq. (S4), we can determine

$$[(\mathbb{E} - \boldsymbol{W})^{-1}]_{jk} = \left[\mathbb{E}^{-\mathbf{1}} \circ \sum_{n=0}^{\infty} \left(\boldsymbol{W} \circ \mathbb{E}^{-\mathbf{1}}\right)^{\boldsymbol{n}}\right]_{jk}$$

$$= \left[\mathbb{E}^{-\mathbf{1}}\right]_{jk} + \sum_{n=1}^{\infty} \sum_{i_1, i_2, \ldots, i_n,} \left[\mathbb{E}^{-\mathbf{1}}\right]_{ji_1} \boldsymbol{W}_{i_1 i_2} \left[\mathbb{E}^{-\mathbf{1}}\right]_{i_2 i_3} \ldots \boldsymbol{W}_{i_{n-1} i_n} \left[\mathbb{E}^{-\mathbf{1}}\right]_{i_n k} \quad (S5)$$

Next, we substitute the above expression into the formulas for $\hat{G}^{SD}(\epsilon)$ and $\hat{G}^{DD}(\epsilon)$. Further considerations involve the summation of infinite series that appear in the formulas for the desired Green's functions. To precisely illustrate this process diagrammatically, let's examine the expression for $\hat{G}^{SD}$

$$\hat{G}^{SD}(\epsilon) = \langle[(\mathbb{E}-\boldsymbol{W})^{-1}]_{11}\rangle_{\mathfrak{R}} = \langle\left[\mathbb{E}^{-\mathbf{1}}\right]_{11}\rangle_{\mathfrak{R}} + \langle\left[\mathbb{E}^{-\mathbf{1}}\circ\boldsymbol{W}\circ\mathbb{E}^{-\mathbf{1}}\right]_{11}\rangle_{\mathfrak{R}} + \langle\left[\mathbb{E}^{-\mathbf{1}}\circ\boldsymbol{W}\circ\mathbb{E}^{-\mathbf{1}}\circ\boldsymbol{W}\circ\mathbb{E}^{-\mathbf{1}}\right]_{11}\rangle_{\mathfrak{R}} + \cdots$$

$$= \epsilon^{-1} + \langle\sum_{i,j}^{N}\left[\mathbb{E}^{-\mathbf{1}}\right]_{1i}\boldsymbol{W}_{ij}\left[\mathbb{E}^{-\mathbf{1}}\right]_{j1}\rangle_{\mathfrak{R}} + \langle\sum_{i,j}^{N}\sum_{k,l}^{N}\left[\mathbb{E}^{-\mathbf{1}}\right]_{1i}\boldsymbol{W}_{ij}\left[\mathbb{E}^{-\mathbf{1}}\right]_{jk}\boldsymbol{W}_{kl}\left[\mathbb{E}^{-\mathbf{1}}\right]_{l1}\rangle_{\mathfrak{R}} + \cdots \qquad \text{(S6)}$$

We transform the first (double) sum in the above equation into the form

$$\sum_{i,j}^{N}\left[\mathbb{E}^{-\mathbf{1}}\right]_{1i}\boldsymbol{W}_{ij}\left[\mathbb{E}^{-\mathbf{1}}\right]_{j1} = \epsilon^{-1}\boldsymbol{W}_{11}\epsilon^{-1} = \epsilon^{-1}\left[\sum_{j=1}^{N} w_{x_j x_1}^{DD}\right]\epsilon^{-1} \qquad \text{(S7)}$$

Similarly, applying the same procedure to the second (quadruple) sum, we obtain

$$\sum_{i,j}^{N}\sum_{k,l}^{N}[\mathbb{E}^{-\mathbf{1}}]_{1i}\boldsymbol{W}_{ij}[\mathbb{E}^{-\mathbf{1}}]_{jk}\boldsymbol{W}_{kl}[\mathbb{E}^{-\mathbf{1}}]_{l1} = \sum_{j}^{N}\sum_{k}^{N}\epsilon^{-1}\boldsymbol{W}_{1j}[\mathbb{E}^{-\mathbf{1}}]_{jk}\boldsymbol{W}_{k1}\epsilon^{-1} = \sum_{j=1}^{N}\epsilon^{-1}\boldsymbol{W}_{1j}\epsilon^{-1}\boldsymbol{W}_{j1}\epsilon^{-1} \qquad \text{(S8)}$$

By further calculating, we observe that the n-th sum in the equation for $\hat{G}^{SD}(\epsilon)$ (not explicitly shown there) involves an n-fold product of $\boldsymbol{W}_{1i_1}\boldsymbol{W}_{i_1 i_2}...\boldsymbol{W}_{i_n 1}$. Using the definition of the $\boldsymbol{W}$ matrix, we can see that each element $\boldsymbol{W}_{ij}$ consists of a single term (when $i = j$) or a sum of N terms $\left(\text{when } i \neq j\right)$. Thus, each n-fold product of $\boldsymbol{W}_{ij}$ terms, with m of them being diagonal ($i = j$), generates $(N-1)^m$ products of n-element transfer rates $w_{x_i x_j}^{DD}$. Summing all these products (as $n \to \infty$) to obtain the corresponding Green function requires a special computational technique. The proper method for this purpose is the diagrammatic method.

Let us introduce a diagrammatic representation, (a graph), to depict the transfer rates $w_{x_i x_j}^{DD}$. In this representation, the vertices of the graph are marked with the donor number, and directed arrows represent the transfer rates $w_{x_i x_j}^{DD}$. We adopt the following convention: continuous arrows $w_{x_i x_j}^{DD}$ represent the transfer rate from donor molecule j to donor molecule i, while two arrows corresponding to $(-w_{x_i x_j}^{DD})$ are drawn with a solid line followed by a dashed line (Fig. S1). The sign ($+$ or $-$) assigned to the arrow $w_{x_i x_j}^{DD}$ indicates an increase or decrease in the probability of the respective transition.

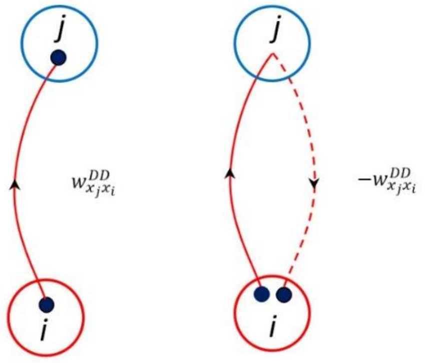

Figure S1: Multigraphs assigned to individual transfers rates $w_{x_i x_j}^{DD}$ and $-w_{x_i x_j}^{DD}$.

In the graph, a path is defined as a sequence of vertices connected by edges, where each vertex is connected to the next vertex in the sequence. Essentially, a path represents a traversal of the graph, moving from one vertex to another along the edges, without revisiting any vertex. Refer to Figure S2 for an illustration.

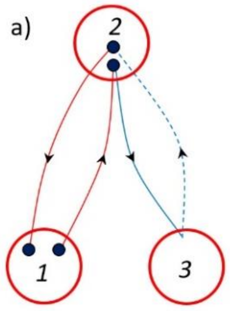

Figure S2: Multigraphs representing paths. a) $w^{DD}_{x_2x_1}(-w^{DD}_{x_3x_2})w^{DD}_{x_1x_2}$ b) $w^{DD}_{x_2x_1}w^{DD}_{x_1x_2}w^{DD}_{x_2x_1}$ c) $(-w^{DD}_{x_2x_1})(-w^{DD}_{x_2x_1})w^{DD}_{x_2x_1}$

A loop in a graph is a path that starts and ends at the same vertex. In the context of the graph representing the transfer rates $w^{DD}_{x_ix_j}$, the multigraphs corresponding to the products $w^{DD}_{x_2x_1}w^{DD}_{x_3x_2}...w^{DD}_{x_{i_n}x_{i_{n-1}}}$ form paths within the graph. These paths can be visualized as sequences of vertices connected by edges, starting and ending at the same vertex. Refer to Figure S3 for an illustration.

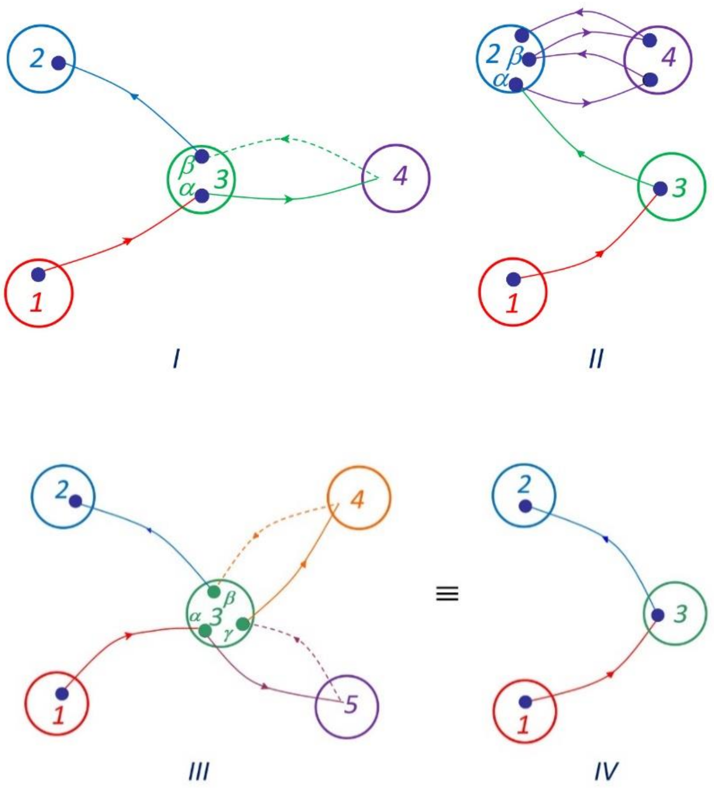

Figure S3: Examples of multigraphs representing $\hat{G}^{DD}(\boldsymbol{k},\epsilon)$ containing loops. Multigraphs (I) and (II) consist of a single loop starting at vertex α and ending at β; multigraph (III) consists of two consecutive loops sharing a common vertex γ. Multigraph (IV) is obtained by removing the loops from (I)-(III).

Let us assign the value ϵ^{-1} to the vertices corresponding to the donors. To each multigraph, a numerical value is assigned calculated by performing an ensemble average of the product formed by a factor of ϵ^{-1} for each donor vertex, a factor of $w^{DD}_{x_ix_j}$ for each solid arrow, and a factor of (-1) for each dashed arrow. The number that we refer to as the value of the multigraph is given by

$$\frac{1}{S^N}\int\limits_S d\boldsymbol{r}_1 \dots \int\limits_S d\boldsymbol{r}_N \prod w_{x_i x_j}^{DD} \prod(-1) \quad \text{(S9)}$$

where $\prod w_{x_i x_j}^{DD}$ and $\prod(-1)$ denote the respective products of factors $w_{x_i x_j}^{DD}$ and the number-1. The factor (-1) appears as many times as there are dashed arrows in the given multigraph. If a given multigraph consists of m circles , the value of such a multigraph does not depend on the numbering of the molecules. As a result, there are $(N-1)(N-2)\dots(N-m)$ numerically equivalent multigraphs. Therefore, we can simplify the diagrammatic series by considering multigraphs with unlabeled vertices and keeping only the topologically inequivalent ones. The value of a topologically inequivalent multigraph is equal to $(N-1)(N-2)\dots(N-m)$ times the value of the corresponding original multigraph. Two multigraphs are considered topologically inequivalent if, after labeling the vertices with the same set of labels, there is no permutation that leads to the equivalence of the multigraphs. To calculate the values of individual multigraphs, we choose an arbitrary representative from each equivalence class and assign fixed labels (any labels) to its vertices. For example, the initially excited donor molecule at $t=0$ is labeled as 1.

Let us consider modifications of diagram IV by assigning the vertex 3 loops with an increasing number of donors, as illustrated in Figure S4.

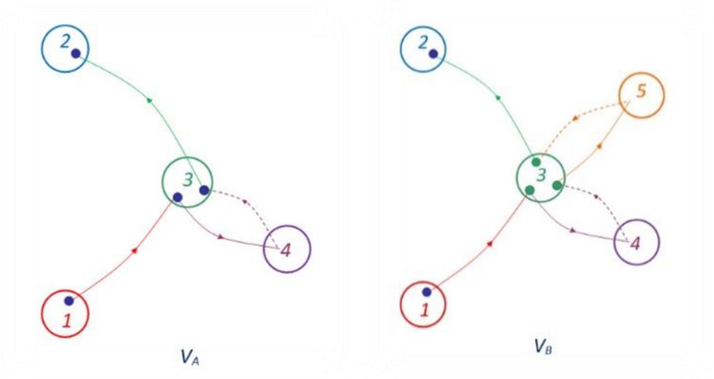

Figure S4: Examples of multigraphs representing $\hat{G}^{DD}(\boldsymbol{k},\epsilon)$ containing loops

The values of the individual multigraphs are as follows

$$V_A = \frac{(N-1)(N-2)(N-3)}{S^3}\int d\boldsymbol{r}_{12}\, exp(i\boldsymbol{k}\boldsymbol{r}_{12}) \int d\boldsymbol{r}_{13} \int d\boldsymbol{r}_{14} \epsilon^{-1} w_{x_3 x_1}^{DD} \epsilon^{-1}\left(-w_{x_4 x_3}^{DD}\right)\epsilon^{-1} w_{x_2 x_3}^{DD} \epsilon^{-1} \quad (S10)$$

$$V_B = \frac{(N-1)(N-2)(N-3)(N-4)}{S^4}\int d\boldsymbol{r}_{12}\, exp(i\boldsymbol{k}\boldsymbol{r}_{12}) \cdot$$

$$\cdot \int d\boldsymbol{r}_{13} \int d\boldsymbol{r}_{14} \int d\boldsymbol{r}_{15} \epsilon^{-1} w_{x_3 x_1}^{DD} \epsilon^{-1}\left(-w_{x_4 x_3}^{DD}\right)\epsilon^{-1}\left(-w_{x_5 x_3}^{DD}\right)\epsilon^{-1} w_{x_2 x_3}^{DD} \epsilon^{-1} \quad (S11)$$

Finally, the diagrammatic series for the Green function $\hat{G}^{SD}(\epsilon)$is as follows: $\hat{G}^{SD}(\epsilon) = \epsilon^{-1} +$ sum of all multigraphs consisting of loops starting in the vertex number *1,* as shown in Figure S5.

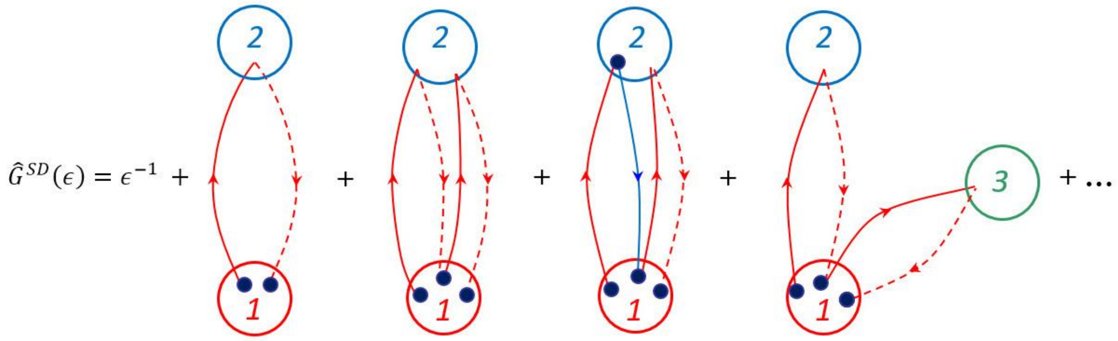

Figure S5: The diagrammatic expansion of the Green's function $\hat{G}^{SD}(\epsilon)$

In a similar manner, we can define a diagrammatic representation of Green function $\hat{G}^{DD}(\boldsymbol{k},\epsilon) =$ sum of all different (topologically) multigraphs consisting of paths starting in a vertex number 1 and ending in a vertex number *2,* as shown in Figure S6.

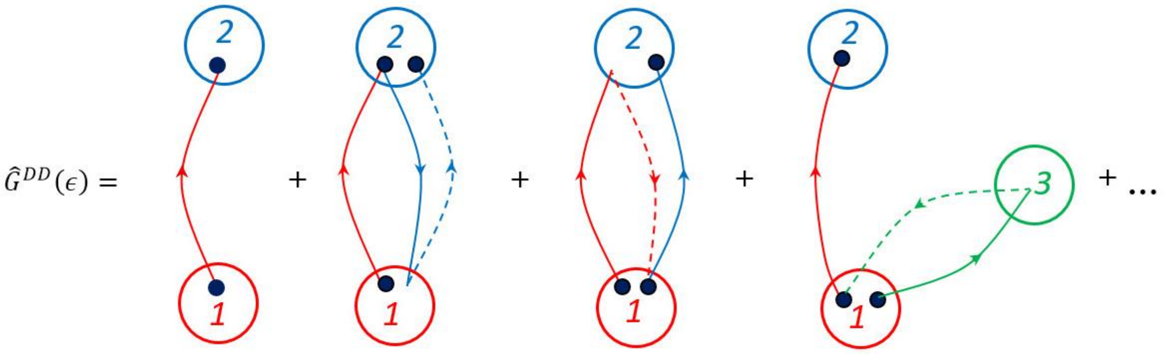

Figure S6: The diagrammatic expansion of the Green's function $\hat{G}^{DD}(\boldsymbol{k},\epsilon)$

We can renormalize the multigraphs containing loops by eliminating the maximum subgraph that forms a loop within the given multigraph. During this process, we merge the start and end vertices of the loop into a single vertex, effectively collapsing the loop. This transformation yields a representative of the class encompassing all multigraphs that share the same donors interconnected by arrows and loops. To compute the value of this transformed multigraph, which no longer contains loops, we make appropriate adjustments to the expressions assigned to its vertices. These modifications accounts for the elimination of the loop and ensures the accurate numerical value of the renormalized multigraph.

If we compare the value of multigraph IV from Fig. S3 (without loops),

$$IV = \frac{(N-1)(N-2)}{\mathrm{S}^2}\int d\boldsymbol{r}_{12}\, exp(i\boldsymbol{k}\boldsymbol{r}_{12})\int d\boldsymbol{r}_{13}\epsilon^{-1}w^{DD}_{x_3x_1}\epsilon^{-1}w^{DD}_{x_2x_3}\epsilon^{-1} \qquad (S12)$$

with the value of multigraph I from the same figure,

$$I = \frac{(N-1)(N-2)}{\mathrm{S}^2}\int d\boldsymbol{r}_{12}\, exp(i\boldsymbol{k}\boldsymbol{r}_{12})\int d\boldsymbol{r}_{13}\epsilon^{-1}w^{DD}_{x_3x_1}\left[\frac{(N-3)}{\mathrm{S}}\int d\boldsymbol{r}_{34}\epsilon^{-1}\left(-w^{DD}_{x_4x_3}\right)\epsilon^{-1}\right]w^{DD}_{x_2x_3}\epsilon^{-1} \qquad (S13)$$

we can observe that multigraph I can be generated from multigraph IV by replacing the factor ϵ^{-1} corresponding to vertex 3 with an expression representing the value of a certain multigraph from the diagrammatic series of $\hat{G}^{SD}(\epsilon)$. This expression is one of the components of the $\hat{G}^{SD}(\epsilon)$ function. The above analysis can be generalized into a theorem stating that the value of any multigraph without loops from the diagrammatic expansion of the Green's function $\hat{G}^{DD}(\boldsymbol{k},\epsilon)$ is computed by assigning to each donor vertex the factor $\hat{G}^{SD}(\epsilon)$ instead of ϵ^{-1}.

The next topological property of the multigraphs in the series representing the Green's function $\hat{G}^{DD}(\boldsymbol{k},\epsilon)$ is the presence of nodes. A node is defined as a vertex in the multigraph that separates it into two disjoint subgraphs. In the renormalized multigraphs (without loops) currently under consideration, each circle (donor) can have at most one node. However, it is possible for a multigraph to contain multiple nodes, with the number of nodes limited by the number of vertices in the multigraph. An example of a multigraph containing nodes is shown in Figure S7.

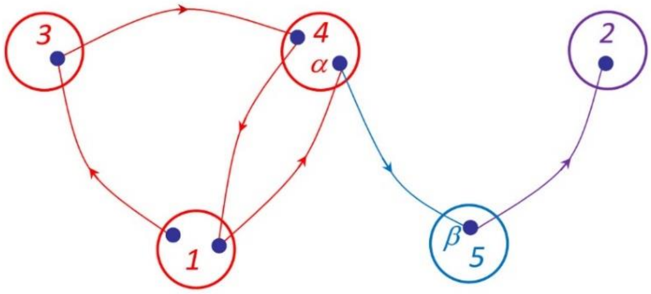

Figure S7: Example of multigraph containing nodes, labeled with Greek letters. After eliminating loops from the multigraphs, we analyze the nodes. In the renormalized multigraphs, each donor (circle) can have at most one node.

If we analyze the topological structure of the multigraph (VI) in Figure S8, we notice that it is the disjoint union of two subgraphs, denoted as (VII) and (VIII), connected at the node labeled as α. The value of the multigraph (VI) is equal to

$$VI(\boldsymbol{k},\epsilon) = \frac{(N-1)(N-2)}{S^2}\left[\hat{G}^{SD}(\epsilon)\right]^3 \int d\boldsymbol{r}_{12}\, exp(i\boldsymbol{k}\boldsymbol{r}_{12}) \int d\boldsymbol{r}_{13}\, w^{DD}_{x_3x_1} w^{DD}_{x_2x_3} =$$

$$= \frac{(N-1)(N-2)}{S^2}\left[\hat{G}^{SD}(\epsilon)\right]^3 \int d\boldsymbol{r}_{13}\, exp(i\boldsymbol{k}\boldsymbol{r}_{13}) w^{DD}_{x_3x_1} \int d\boldsymbol{r}_{32} exp(i\boldsymbol{k}\boldsymbol{r}_{32})\, w^{DD}_{x_2x_3} \qquad \text{(S14)}$$

The values of the multigraphs (VII) and (VIII) are respectively

$$VII(\boldsymbol{k},\epsilon) = \frac{(N-1)}{S}\left[\hat{G}^{SD}(\epsilon)\right]^2 \int d\boldsymbol{r}_{13}\, exp(i\boldsymbol{k}\boldsymbol{r}_{13})\, w^{DD}_{x_3x_1} \qquad \text{(S15)}$$

$$VIII(\boldsymbol{k},\epsilon) = \frac{(N-1)}{S}\left[\hat{G}^{SD}(\epsilon)\right]^2 \int d\boldsymbol{r}_{32}\, exp(i\boldsymbol{k}\boldsymbol{r}_{32})\, w^{DD}_{x_2x_3} \qquad \text{(S16)}$$

Let us note that the value of the multigraph (VI) consisting of three vertices is equal to the product of the values of its subgraphs (VII) and (VIII) with two vertices. However, to the node α, which was formed by identifying the final vertex of the first subgraph (VII) with the initial vertex of the second subgraph (VIII), we now assign the factor $1/\hat{G}^{SD}(\epsilon)$.

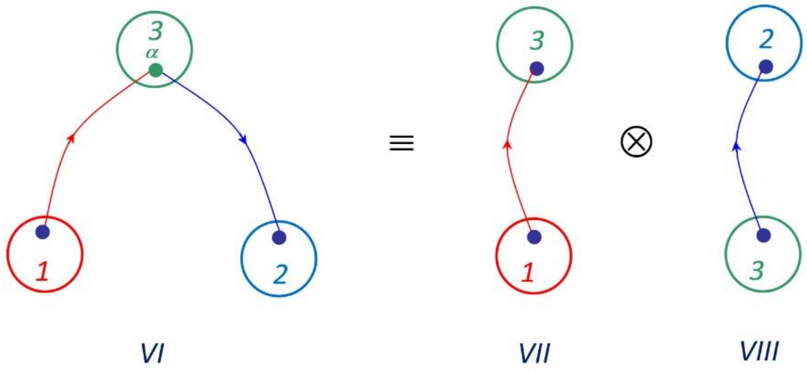

Figure S8: Multigraphs from which the multigraph (VI) can be constructed. The multigraph (VI) is the topological sum of two multigraphs (VII) and (VIII), which are its subgraphs connected at the node α. The value of the multigraph (VI) is equal to the product of the values of its subgraphs (VII) and (VIII), with the node α assigned the factor $1/\hat{G}^{SD}(\epsilon)$.

In summary, the value of any multigraph (without loops) containing nodes can be approximated by multiplying the values of its individual subgraphs, to which it is divided. Notably, we assign the factor $1/\hat{G}^{SD}(\epsilon)$ to the donor nodes.

Continuing the analysis of the diagrammatic representation of the Green's function $\hat{G}^{DD}(\boldsymbol{k}, \epsilon)$, which can contain any number of nodes (but without loops), let us express the function $\hat{G}^{DD}(\boldsymbol{k}, \epsilon)$ as follows

$$\hat{G}^{DD}(\boldsymbol{k}, \epsilon) = \sum_{n=0}^{\infty} \hat{G}_n^{DD}\left(\boldsymbol{k}, \epsilon, \hat{G}^{SD}(\epsilon)\right) \tag{S17}$$

Here, the function $\hat{G}_n^{DD}\left(\boldsymbol{k}, \epsilon, \hat{G}^{SD}(\epsilon)\right)$ represents the sum of all loop-free multigraphs from the series in $\hat{G}^{DD}(\boldsymbol{k}, \epsilon)$ that contain n nodes.

The method of calculating the values of multigraphs suggests the introduction of the following fundamental series: $\hat{\Sigma}^{DD}\left(\boldsymbol{k}, \epsilon, \hat{G}^{SD}(\epsilon)\right)$, which represents the sum of all multigraphs without loops and without nodes starting from specific donor (e.g., donor 1) and ending at a different specified donor (e.g., donor 2). Let's denote the numerical value of this series as Σ^{DD}.

Furthermore, we can denote

$$\Sigma_*^{DD} = \frac{\Sigma^{DD}}{\hat{G}^{SD}(\epsilon)} \tag{S18}$$

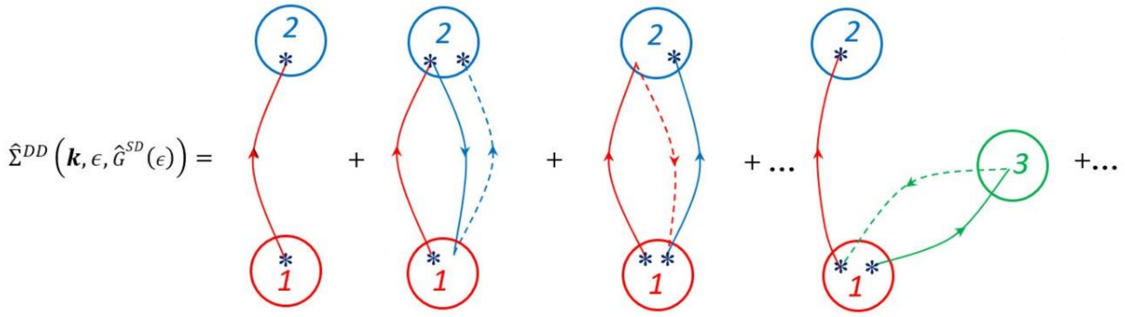

Figure S9: The simplest multigraphs from the diagrammatic series $\hat{\Sigma}^{DD}\left(\boldsymbol{k},\epsilon,\hat{G}^{SD}(\epsilon)\right)$. They do not contain loops and nodes. The vertices corresponding to the donors (circles) are marked with the symbol (*) to indicate that they are assigned to the function $\hat{G}^{SD}(\epsilon)$ after eliminating the loops. Multigraphs containing two donors are referred to as two-body multigraphs, those containing three donors are called three-body multigraphs, and so on.

When calculating the individual terms of the series in $\hat{G}^{DD}(\boldsymbol{k},\epsilon)$, we can express them as follows: $\hat{G}_0^{DD} = \Sigma^{DD}$, $\hat{G}_1^{DD} = \Sigma_*^{DD}\Sigma^{DD}$, $\hat{G}_2^{DD} = \Sigma_*^{DD}\Sigma_*^{DD}\Sigma^{DD}$, and so on, where each term can be seen as forming an infinite geometric series. By summing this series, we obtain:

$$\hat{G}^{DD}(\boldsymbol{k},\epsilon) = \sum_{n=0}^{\infty} \hat{G}_n^{DD}\left(\boldsymbol{k},\epsilon,\hat{G}^{SD}(\epsilon)\right) = \frac{\Sigma^{DD}}{1-\Sigma_*^{DD}} \equiv \frac{\hat{\Sigma}^{DD}}{1-\frac{\hat{\Sigma}^{DD}}{\hat{G}^{SD}}} \qquad (S19)$$

Following the previous analysis, we have determined that knowing the values of the diagrammatic series $\hat{\Sigma}^{DD}\left(\boldsymbol{k},\epsilon,\hat{G}^{SD}(\epsilon)\right)$ is sufficient for determining the time decays and stationary values of the observables in the system. However, this series cannot be exactly summed, and we must rely on approximate methods. In the two-body approximation, we calculate the function $\hat{\Sigma}^{DD}\left(\boldsymbol{k},\epsilon,\hat{G}^{SD}(\epsilon)\right)$only for two-body multigraphs. Similarly, in the three-body approximation, we consider both two-body and three-body multigraphs. To obtain results for the fundamental observables, we apply the self-consistent method. This involves finding an expression for $\hat{\Sigma}^{DD}\left(\boldsymbol{k},\epsilon,\hat{G}^{SD}(\epsilon)\right)$ in the appropriate approximation and treating the function $\hat{G}^{SD}(\epsilon)$ as the variable we are searching for. We determine this variable by substituting the Green's function $\hat{G}^{DD}(\boldsymbol{k},\epsilon)$, which depends on $\hat{\Sigma}^{DD}\left(\boldsymbol{k},\epsilon,\hat{G}^{SD}(\epsilon)\right)$, into the self-consistent equation $\hat{G}^{SD}(\epsilon) + \hat{G}^{DD}(\boldsymbol{k}=\boldsymbol{0},\epsilon) = \epsilon^{-1}$. This self-consistent procedure greatly improves the accuracy of calculations and is simpler compared to other methods that involve approximating both the functions $\hat{G}^{SD}(\epsilon)$ and $\hat{G}^{DD}(\boldsymbol{k},\epsilon)$ using appropriate expansions into diagrammatic series.

TWO- AND THREE-BODY APPROXIMATIONS: GENERAL FORMULAS.

As derived from the previous chapter, to obtain the time decays and stationary values of observables characterizing the studied system, knowledge of the values of the diagrammatic series $\hat{\Sigma}^{DD}\left(\boldsymbol{k},\epsilon,\hat{G}^{SD}(\epsilon)\right)$ is sufficient. Unfortunately, this series cannot be precisely summed, necessitating the use of approximate methods. The two-body approximation involves restricting calculations of the function $\hat{\Sigma}^{DD}\left(\boldsymbol{k},\epsilon,\hat{G}^{SD}(\epsilon)\right)$ exclusively to two-body multigraphs. Similarly, in the three-body approximation, we only consider two-body and three-body multigraphs (refer to Figure S9). The two-

body approximation relies on a rigorous solution to the two-body problem. To achieve this, we expand the Green function $\hat{G}^{DD}(\boldsymbol{k},\epsilon)$ in a series with respect to the concentration of molecules for a system consisting of N donors positioned on a sphere with a radius R and surface S

$$\hat{G}^{DD}(\boldsymbol{k},\epsilon,N)=\frac{(N-1)}{S}C_2^{DD}(\boldsymbol{k},\epsilon)+\frac{(N-1)(N-2)}{S^2}C_3^{DD}(\boldsymbol{k},\epsilon)+$$
$$+\frac{(N-1)(N-2)(N-3)}{S^3}C_4^{SD}(\boldsymbol{k},\epsilon)+\cdots \tag{S20}$$

Based on the equation provided, we can conclude that

$$C_2^{DD}(\boldsymbol{k},\epsilon)=S\,\hat{G}^{DD}(\boldsymbol{k},\epsilon,N=2)=S\,\langle exp(i\boldsymbol{k}\boldsymbol{r}_{12})[(\mathbb{E}-\boldsymbol{W})^{-1}]_{21}\rangle_{\Re} \tag{S21}$$

The function $C_2^{DD}(\boldsymbol{k},\epsilon)$, representing two-body multigraphs, corresponds to multigraphs without nodes, as a system of two molecules cannot have nodes. These multigraphs may contain loops. Therefore, to obtain the desired expression for the series $\hat{\Sigma}_2^{DD}\left(\boldsymbol{k},\epsilon,\hat{G}^{SD}(\epsilon)\right)$, we simply need to replace $\epsilon^{-1}$with the function $\hat{G}^{SD}(\epsilon)$ in Eq. (S21).
The explicit form of the $\mathbb{E}-\boldsymbol{W}$ matrix for the considered system of molecules with $N=2$ is as follows:

$$\mathbb{E}-\boldsymbol{W}=\begin{vmatrix}\epsilon+w_{x_2x_1}^{DD} & -w_{x_1x_2}^{DD}\\ -w_{x_2x_1}^{DD} & \epsilon+w_{x_1x_2}^{DD}\end{vmatrix} \tag{S22}$$

Next, when calculating the elements of the inverse matrix, we obtain:

$$[(\mathbb{E}-\boldsymbol{W})^{-1}]_{21}=\frac{w_{x_2x_1}^{DD}}{det(\mathbb{E}-\boldsymbol{W})}=\frac{\epsilon^{-2}\,w_{x_2x_1}^{DD}}{1+2\,\epsilon^{-1}w_{x_2x_1}^{DD}} \tag{S23}$$

where we used the transfer rates symmetry, $w_{x_2x_1}^{DD}=w_{x_1x_2}^{DD}$.
By averaging over the configuration of molecules, and removing loops, $\epsilon^{-1}\to\hat{G}^{SD}(\epsilon)$, we obtain:

$$\hat{\Sigma}_2^{DD}\left(\boldsymbol{k},\epsilon,\hat{G}^{SD}(\epsilon)\right)=\frac{N-1}{S}\int d\boldsymbol{r}_{12}\,exp(i\boldsymbol{k}\boldsymbol{r}_{12})\frac{\left(\hat{G}^{SD}\right)^2w_{x_2x_1}^{DD}}{1+2\,\hat{G}^{SD}w_{x_2x_1}^{DD}} \tag{S24}$$

The function $\hat{\Sigma}_3^{DD}\left(\boldsymbol{k},\epsilon,\hat{G}^{SD}(\epsilon)\right)$, which represents the three-body approximation for $\hat{\Sigma}^{DD}\left(\boldsymbol{k},\epsilon,\hat{G}^{SD}(\epsilon)\right)$, is defined as the sum of the values of all multigraphs without nodes and without loops, formed by three donors. The most straightforward approach to calculate it is to first determine the sum of all three-body multigraphs $\hat{\mathrm{A}}_3^{DDD}(\boldsymbol{k},\epsilon)$, and then subtract from it the multigraphs with loops $\hat{\mathrm{L}}_3^{DDD}(\boldsymbol{k},\epsilon)$ and nodes $\hat{\mathrm{N}}_3^{DDD}(\boldsymbol{k},\epsilon)$. After performing the renormalization, $\epsilon^{-1}\to\hat{G}^{SD}(\epsilon)$, we obtain the expression for the function $\hat{\Sigma}_3^{DD}\left(\boldsymbol{k},\epsilon,\hat{G}^{SD}(\epsilon)\right)$.

MULTIGRAPHS $\hat{\mathrm{A}}_3^{DDD}(\boldsymbol{k},\epsilon)$

To sum the values of all three-body multigraphs $\hat{\mathrm{A}}_3^{DDD}(\boldsymbol{k},\epsilon)$, we once again utilize the expansion in Eq. (S20) which represents the expansion of the Green's function $\hat{G}^{DD}(\boldsymbol{k},\epsilon)$ in a power series with respect to the concentration of donors on a sphere with a radius R and surface S. By interpreting the individual components of this series, we can conclude that the function $C_3^{DD}(\boldsymbol{k},\epsilon)$ corresponds to the three-body multigraphs $\hat{\mathrm{A}}_3^{DDD}(\boldsymbol{k},\epsilon)$. By substituting the appropriate value for N, we obtain

$$\hat{G}^{DD}(\boldsymbol{k},\epsilon,N=3)=\frac{2}{S}C_2^{DD}(\boldsymbol{k},\epsilon)+\frac{2}{S^2}C_3^{DD}(\boldsymbol{k},\epsilon) \tag{S25}$$

Using the definition of the Green's function $\hat{G}^{DD}(\boldsymbol{k},\epsilon)$, we obtain

$$C_3^{DD}(\boldsymbol{k},\epsilon)=S^2\,\langle exp(i\boldsymbol{k}\boldsymbol{r}_{13})[(\mathbb{E}-\boldsymbol{W})^{-1}]_{31}\rangle_{\Re}-S\,C_2^{DD}(\boldsymbol{k},\epsilon) \tag{S26}$$

To compute $C_3^{DD}(\boldsymbol{k},\epsilon)$, we consider the $\mathbb{E}-\boldsymbol{W}$ matrix that appears in the definition of the Green's function $\hat{G}^{DD}(\boldsymbol{k},\epsilon,N=3)$). In this case, the $\mathbb{E}-\boldsymbol{W}$ matrix is a $3x3$ matrix, and it takes the following form

$$\mathbb{E}-\boldsymbol{W}=\begin{vmatrix}\epsilon+w_{x_2x_1}^{DD}+w_{x_3x_1}^{DD} & -w_{x_1x_2}^{DD} & -w_{x_1x_3}^{DD}\\ -w_{x_2x_1}^{DD} & \epsilon+w_{x_1x_2}^{DD}+w_{x_3x_2}^{DD} & -w_{x_2x_3}^{DD}\\ -w_{x_3x_1}^{DD} & -w_{x_3x_2}^{DD} & \epsilon+w_{x_1x_3}^{DD}+w_{x_2x_3}^{DD}\end{vmatrix} \tag{S27}$$

Using Eq. (S27), we obtain

$$[(\mathbb{E}-\boldsymbol{W})^{-1}]_{31}=\frac{det\begin{vmatrix}-w_{x_2x_1}^{DD} & \epsilon+w_{x_1x_2}^{DD}+w_{x_3x_2}^{DD}\\ -w_{x_3x_1}^{DD} & -w_{x_3x_2}^{DD}\end{vmatrix}}{det(\epsilon\,\mathbb{I}-\boldsymbol{W})}\equiv A(\boldsymbol{r}_{12},\boldsymbol{r}_{13},\epsilon) \tag{S28}$$

Based on Eq. (S26) and (S28), we can derive the final result as follows

$$C_3^{DD}(\boldsymbol{k},\epsilon)=\int d\boldsymbol{r}_{12}\int d\boldsymbol{r}_{13}\,exp(i\boldsymbol{k}\boldsymbol{r}_{13})\left\{A(\boldsymbol{r}_{12},\boldsymbol{r}_{13},\epsilon)-\frac{\epsilon^{-2}w_{x_2x_1}^{DD}}{1+2\,\epsilon^{-1}w_{x_2x_1}^{DD}}\right\} \tag{S29}$$

After applying the renormalization procedure, $\epsilon^{-1}\to\hat{G}^{SD}(\epsilon)$, to the above formula, we obtain the equation that describes the desired function $\hat{A}_3^{DDD}(\boldsymbol{k},\epsilon)$

$$\hat{A}_3^{DDD}(\boldsymbol{k},\epsilon)=\frac{(N-1)(N-2)}{S^2}\int d\boldsymbol{r}_{12}\,exp(i\boldsymbol{k}\boldsymbol{r}_{12})\int d\boldsymbol{r}_{13}\left\{A(\boldsymbol{r}_{12},\boldsymbol{r}_{13},\hat{G}^{SD})-\frac{(\hat{G}^{SD})^2w_{x_2x_1}^{DD}}{1+2\hat{G}^{SD}w_{x_2x_1}^{DD}}\right\} \tag{S30}$$

The function $A(\boldsymbol{r}_{12},\boldsymbol{r}_{13},\hat{G}^{SD})=A_L(\boldsymbol{r}_{12},\boldsymbol{r}_{13},\hat{G}^{SD})/A_M(\boldsymbol{r}_{12},\boldsymbol{r}_{13},\hat{G}^{SD})$ is given by

$$A_L(\boldsymbol{r}_{12},\boldsymbol{r}_{13},\hat{G}^{SD})=(\hat{G}^{SD})^2[w_{x_2x_1}^{DD}+\hat{G}^{SD}(w_{x_2x_1}^{DD}w_{x_3x_1}^{DD}+w_{x_2x_1}^{DD}w_{x_3x_2}^{DD}+w_{x_3x_1}^{DD}w_{x_3x_2}^{DD})] \tag{S31}$$

$$A_M(\boldsymbol{r}_{12},\boldsymbol{r}_{13},\hat{G}^{SD})=1+2\,\hat{G}^{SD}(w_{x_2x_1}^{DD}+w_{x_3x_1}^{DD}+w_{x_3x_2}^{DD})+ \\ +3\,(\hat{G}^{SD})^2(w_{x_2x_1}^{DD}w_{x_3x_2}^{DD}+w_{x_2x_1}^{DD}w_{x_3x_1}^{DD}+w_{x_3x_1}^{DD}w_{x_3x_2}^{DD}) \tag{S32}$$

When deriving the above formulas, we used the transfer rates symmetry, $w_{x_ix_j}^{DD}=w_{x_jx_i}^{DD}$.

MULTIGRAPHS $\hat{L}_3^{DDD}(\boldsymbol{k},\epsilon)$

The sum of the values of all three-body multigraphs with loops $\hat{L}_3^{DDD}(\boldsymbol{k},\epsilon)$ is determined using the principle of mathematical induction. This involves adding together their respective values in a standard

manner. The multigraphs under consideration, which consist of three donors, are illustrated in Figure S10.

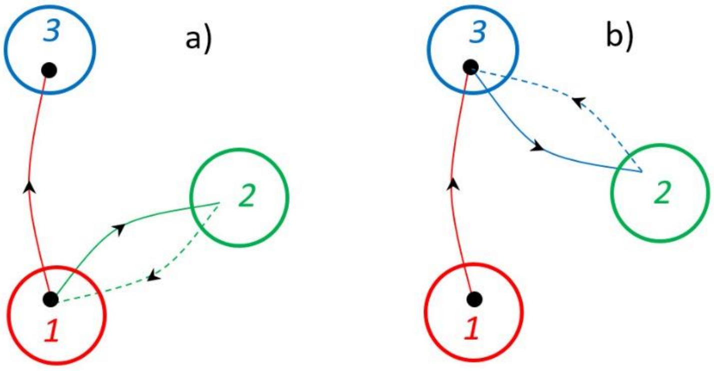

Fig. S10. The multigraphs representing $\hat{\mathrm{L}}_3^{DDD}(\boldsymbol{k},\epsilon)$ correspond to a system of three molecules, allowing for one loop. The loop can start in either donor number 1 (a) or donor number 3 (b).

These multigraphs can consist of a loop starting in either donor 1 or donor 3, and they have the same value. Upon analyzing the topological structure of the multigraphs in the series $\hat{\mathrm{L}}_3^{DDD}(\boldsymbol{k},\epsilon)$, we observe that they have the same form as the generating multigraphs $\hat{\Sigma}_2^{DD}(\boldsymbol{k},\epsilon)$. However, the number of multigraphs differs, and the donor nodes are assigned the function $B_2^{SD}(\epsilon)$ instead of ϵ^{-1}. Here, $B_2^{SD}(\epsilon)$ represents a linear component in the diagrammatic expansion of the function $\hat{G}^{SD}(\epsilon)$

$$\hat{G}^{SD}(\epsilon)=\epsilon^{-1}+\frac{N-1}{S}B_2^{SD}(\epsilon)+\frac{(N-1)(N-2)}{S^2}B_3^{SD}(\epsilon)+\cdots\ , \tag{S33}$$

which is expanded in powers of the donor concentration (i.e., the numerical value of the loop being considered).

Let's define a series $\hat{\Sigma}_{2L}^{DD,n}\left(\boldsymbol{k},\epsilon,B_2^{SD}(\epsilon)\right)$ as the sum of all multigraphs starting in donor 1 and ending in donor 3, containing n continuous arrows, and having one loop in the set of donor vertices

$$\hat{\mathrm{L}}_{3A}^{DDD}(\boldsymbol{k},\epsilon)=\sum_{n=1}^{\infty}\hat{\Sigma}_{2L}^{DD,n}\left(\boldsymbol{k},\epsilon,B_2^{SD}(\epsilon)\right) \tag{S34}$$

where the function $\hat{\mathrm{L}}_{3A}^{DDD}(\boldsymbol{k},\epsilon)$ refers to the case illustrated on the left side of Fig. S10. We divide the multigraphs $\hat{\Sigma}_{2L}^{DD,n}\left(\boldsymbol{k},\epsilon,B_2^{SD}(\epsilon)\right)$ into subclasses $\hat{\Sigma}_{2L,l,m}^{DD,n}\left(\boldsymbol{k},\epsilon,B_2^{SD}(\epsilon)\right)$, which consist of l continuous arrows from donor 1 to donor 3 and m continuous arrows from donor 3 to donor 1 ($n=l+m$). Each donor vertex in this subclass (of which there are l) can be assigned a loop, but only one for a given set of vertices. Therefore, when analyzing individual multigraphs from the class $\hat{\Sigma}_{2L,l,m}^{DD,n}$, it can be observed that for a fixed l, ($m=n-l$), there exist $\binom{n-1}{m}\equiv\binom{n-1}{n-l}=\binom{n-1}{l-1}$ numerically equivalent multigraphs with the following value

$$\hat{\Sigma}_{2L,l,m}^{DD,n}\left(\boldsymbol{k},\epsilon,B_2^{SD}(\epsilon)\right)=(-1)^{n+1}\,\frac{(N-1)(N-2)}{S^2}B_2^{SD}(\epsilon)\int d\boldsymbol{r}_{13}\,exp(i\boldsymbol{k}\boldsymbol{r}_{13})\left(\epsilon^{-1}w_{x_3x_1}^{DD}\right)^l\left(\epsilon^{-1}w_{x_1x_3}^{DD}\right)^{n-l} \tag{S35}$$

From the above, it follows that

$$\hat{\Sigma}_{2L}^{DD,n}\left(\boldsymbol{k},\epsilon,B_2^{SD}(\epsilon)\right)=\sum_{l=1}^{n} l\binom{n-1}{l-1}\hat{\Sigma}_{2L,l,m}^{DD,n}\left(\boldsymbol{k},\epsilon,B_2^{SD}(\epsilon)\right) \qquad (S36)$$

By using the identity

$$\sum_{l=1}^{n} l\binom{n-1}{l-1}\left(\epsilon^{-1}w_{x_3x_1}^{DD}\right)^{l}\left(\epsilon^{-1}w_{x_1x_3}^{DD}\right)^{n-l}=\epsilon^{-n}w_{x_3x_1}^{DD}\left(n\,w_{x_3x_1}^{DD}+w_{x_1x_3}^{DD}\right)\left(w_{x_3x_1}^{DD}+w_{x_1x_3}^{DD}\right)^{n-2} \quad (S37)$$

and summing up the values of multigraphs $\hat{\Sigma}_{2L}^{DA,n}\left(\boldsymbol{k},\epsilon,B_2^{SD}(\epsilon)\right)$, according to Eq. (S34), we obtain the first component of the function $\hat{\mathrm{L}}_{3A}^{DDA}(\boldsymbol{k},\epsilon)$

$$\hat{\mathrm{L}}_{3A}^{DDD}(\boldsymbol{k},\epsilon)=\frac{(N-1)(N-2)}{S^2}B_2^{SD}(\epsilon)\int d\boldsymbol{r}_{13}exp(i\boldsymbol{k}\boldsymbol{r}_{13})\frac{\epsilon^{-1}w_{x_3x_1}^{DD}\left(1+\epsilon^{-1}w_{x_1x_3}^{DD}\right)}{\left(1+\epsilon^{-1}w_{x_3x_1}^{DD}+\epsilon^{-1}w_{x_1x_3}^{DD}\right)^2} \qquad (S38)$$

where we used the formula

$$\sum_{n=1}^{\infty}(-1)^{n+1}\epsilon^{-n}\left(n\,w_{x_3x_1}^{DD}+w_{x_1x_3}^{DD}\right)\left(w_{x_3x_1}^{DD}+w_{x_1x_3}^{DD}\right)^{n-2}=\frac{\epsilon^{-1}w_{x_3x_1}^{DD}\left(1+\epsilon^{-1}w_{x_1x_3}^{DD}\right)}{\left(1+\epsilon^{-1}w_{x_3x_1}^{DD}+\epsilon^{-1}w_{x_1x_3}^{DD}\right)^2} \quad (S39)$$

The value of multigraphs consisting of a loop starting in donor 3 is the same as $\hat{\mathrm{L}}_{3A}^{DDD}(\boldsymbol{k},\epsilon)$. The sum of the values of all three-body multigraphs with loops, $\hat{\mathrm{L}}_{3}^{DDD}(\boldsymbol{k},\epsilon)$, can be obtained by using the symmetry of the transfer rates $w_{x_3x_1}^{DD}=w_{x_1x_3}^{DD}$. After the renormalization procedure, $\epsilon^{-1}\rightarrow\hat{G}^{SD}(\epsilon)$, the result is as follows

$$\hat{\mathrm{L}}_{3}^{DDD}(\boldsymbol{k},\epsilon)=2\,\frac{(N-1)(N-2)}{S^2}B_2^{SD}(\epsilon)\int d\boldsymbol{r}_{13}\,exp(i\boldsymbol{k}\boldsymbol{r}_{13})\frac{\hat{G}^{SD}(\epsilon)w_{x_3x_1}^{DD}\left(1+\hat{G}^{SD}(\epsilon)w_{x_3x_1}^{DD}\right)}{\left(1+2\,\hat{G}^{SD}(\epsilon)w_{x_3x_1}^{DD}\right)^2} \quad (S40)$$

The function $B_2^{SD}(\epsilon)$ in Eq. (S38) is calculated from the diagrammatic expansion of the function $\hat{G}^{SD}(\epsilon)$, Eq. (S33), as follows: $B_2^{SD}(\epsilon)=S\left(\hat{G}^{SD}(\epsilon,N=2)-\epsilon^{-1}\right)=S(\langle[(\mathbb{E}-\boldsymbol{W})^{-1}]_{11}\rangle-\epsilon^{-1})$. Performing calculations similar to those used when calculating $C_2^{DD}(\boldsymbol{k},\epsilon)$, we finally obtain the expression for $B_2^{SD}(\epsilon)$ as follows

$$B_2^{SD}(\epsilon)=-\int d\boldsymbol{r}_{12}\frac{\left(\hat{G}^{SD}\right)^2w_{x_2x_1}^{DD}}{\left(1+2\,\hat{G}^{SD}w_{x_2x_1}^{DD}\right)} \qquad (S41)$$

MULTIGRAPHS $\hat{\mathrm{N}}_3^{DDD}$

As it is easy to see for a system of three donors, there can only be one node (no loops). This situation is depicted in Fig. S11.

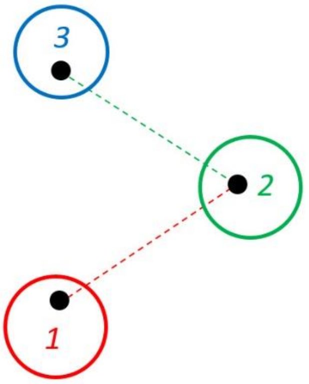

Figure S11. Three-body multigraphs representing the function $\hat{N}_3^{DDD}(\boldsymbol{k},\epsilon)$. They can only contain one node.

The sum of this diagrammatic series is obtained by multiplying the values of the relevant multigraphs from donor 1 to the node and from the node (in donor 2) and from the node to donor 3. After a simple analysis, we obtain.

$$\hat{N}_3^{DDD}\left(\boldsymbol{k},\epsilon,\hat{G}^{SD}(\epsilon)\right)=\frac{\hat{\Sigma}_2^{DD}\left(\boldsymbol{k},\epsilon,\hat{G}^{SD}(\epsilon)\right)\hat{\Sigma}_2^{DD}\left(\boldsymbol{k},\epsilon,\hat{G}^{SD}(\epsilon)\right)}{\hat{G}^{SD}(\epsilon)} \quad (S42)$$

Substituting explicit expressions on two-body functions $\hat{\Sigma}_2^{DD}\left(\boldsymbol{k},\epsilon,\hat{G}^{SD}(\epsilon)\right)$ Eq. (S42) becomes:

$$\hat{N}_3^{DDD}\left(\boldsymbol{k},\epsilon,\hat{G}^{SD}(\epsilon)\right)=\frac{(N-1)^2}{S^2}\left(\hat{G}^{SD}\right)^3\int d\boldsymbol{r}_{12}\int d\boldsymbol{r}_{13}\,exp(i\boldsymbol{k}\boldsymbol{r}_{13})\,N\left(\boldsymbol{r}_{12},\boldsymbol{r}_{13},\hat{G}^{SD}\right) \quad (S43)$$

where

$$N\left(\boldsymbol{r}_{12},\boldsymbol{r}_{13},\hat{G}^{SD}\right)=\frac{w_{x_2x_1}^{DD}}{1+2\,\hat{G}^{SD}w_{x_2x_1}^{DD}}\cdot\frac{w_{x_3x_2}^{DD}}{1+2\,\hat{G}^{SD}w_{x_3x_2}^{DD}} \quad (S44)$$

Supporting Information B: The multigraphs series for two- and three-body approximation

B.1. 2-body approximation

In the two-body approximation adapted for finite-volume system, the function $\hat{\Sigma}^{DD}\left(\boldsymbol{k},\epsilon,\hat{G}^{SD}(\epsilon)\right)$

$$\hat{\Sigma}_2^{DD}\left(\boldsymbol{0},\epsilon,\hat{G}^{SD}(\epsilon)\right)=\frac{N-1}{S}\int d\boldsymbol{r}_{12}\frac{\left(\hat{G}^{SD}\right)^2 w_{x_2x_1}^{DD}}{1+2\,\hat{G}^{SD}w_{x_2x_1}^{DD}} \quad (S45)$$

can be calculated using spherical coordinates. If we denote the angle between the vectors $\boldsymbol{r}_1$ and $\boldsymbol{r}_2$ ($\boldsymbol{r}_{12}=\boldsymbol{r}_2-\boldsymbol{r}_1$), as θ, we can use the trigonometric relationship on a sphere of radius R, $r_{12}=\sqrt{2R^2(1-cos\theta)}$, and by substituting $x=sin^2\left(\frac{\theta}{2}\right)$, we obtain

$$\hat{\Sigma}_2^{DD}\left(\boldsymbol{0},\epsilon,\hat{G}^{SD}(\epsilon)\right)=(N-1)\frac{\left(\hat{G}^{SD}\right)^2}{\tau_{0D}}\left(\frac{R_0^{DD}}{2R}\right)^6\int_0^1\frac{dx}{x^3+2\,\frac{\hat{G}^{SD}}{\tau_{0D}}\left(\frac{R_0^{DD}}{2R}\right)^6} \quad (S46)$$

This integral can be computed analytically to obtain

$$\hat{\Sigma}_2^{DD}\left(\mathbf{0},\epsilon,\hat{G}^{SD}(\epsilon)\right)=(N-1)\frac{\hat{G}^{SD}\,a}{12}\left\{ln\left|\frac{(1+a)^2}{1-a+a^2}\right|+2\sqrt{3}\,arctg\left(\frac{2-a}{a\sqrt{3}}\right)+\sqrt{3}\frac{\pi}{6}\right\} \quad (S47)$$

where

$$a=\left(\frac{R_0^{DD}}{2R}\right)^2\left(\frac{2\,\hat{G}^{SD}}{\tau_{0D}}\right)^{1/3} \quad (S48)$$

For $R_0^{DD}<R$, meaning for very small values of a, we can derive the following approximate formula by using the expansions of the logarithm and arctangent functions in series

$$\hat{\Sigma}_2^{DD}\left(\mathbf{0},\epsilon,\hat{G}^{SD}(\epsilon)\right)\approx(N-1)\frac{\hat{G}^{SD}}{36}\sqrt[3]{2}\,\sqrt{3}\,\pi\left(\frac{R_0^{DD}}{R}\right)^2\left(\frac{\hat{G}^{SD}}{\tau_{0D}}\right)^{\frac{1}{3}} \quad (S49)$$

B.2. 3-body approximation

The sum of all $\widehat{A}_3^{DDD}(\boldsymbol{k},\epsilon)$ three-body multigraphs defined in the Eq. (14) can be written as

$$\widehat{A}_3^{DDD}(\boldsymbol{k}=\mathbf{0},\epsilon)=(N-1)(N-2)\,f_3\left(\hat{G}^{SD}\right) \quad (S50)$$

where

$$f_3\left(\hat{G}^{SD}\right)=\frac{1}{S^2}\int d\boldsymbol{r}_{12}\int d\boldsymbol{r}_{13}\left\{A(\boldsymbol{r}_{12},\boldsymbol{r}_{13},\epsilon)-\frac{\left(\hat{G}^{SD}\right)^2 w_{x_2x_1}^{DD}}{1+2\,\hat{G}^{SD}w_{x_2x_1}^{DD}}\right\} \quad (S51)$$

The numerical value of the function $f_3\left(\hat{G}^{SD}\right)$ is obtained by applying numerical integration in spherical coordinates on a sphere with a radius R. It can be expressed as

$$f_3\left(\hat{G}^{SD}\right)=\frac{1}{16\pi^2}\iint_0^{\pi}d\vartheta_1 d\vartheta_2\,sin\vartheta_1 sin\vartheta_2\iint_0^{2\pi}d\varphi_1 d\varphi_2\left\{A(\vartheta_1,\vartheta_2,\varphi_1,\varphi_2,\epsilon)-\frac{\left(\hat{G}^{SD}\right)^2 w_{x_2x_1}^{DD}}{1+2\,\hat{G}^{SD}w_{x_2x_1}^{DD}}\right\} \quad (S52)$$

Using geometric relationships on a sphere with radius R

$$r_{12}^2=2R^2(1-cos\vartheta_1),\quad r_{13}^2=2R^2(1-cos\vartheta_2) \quad (S53)$$

$$r_{23}^2=2R^2\left(1-cos\vartheta_1 cos\vartheta_2-sin\vartheta_1 sin\vartheta_2 cos(\varphi_2-\varphi_1)\right) \quad (S54)$$

and using substitution

$$x=4\,\frac{\left(\frac{\hat{G}^{SD}}{\tau_{0D}}\right)^{-\frac{1}{3}}}{\xi^2}sin^2\left(\frac{\vartheta_1}{2}\right),\qquad y=4\,\frac{\left(\frac{\hat{G}^{SD}}{\tau_{0D}}\right)^{-\frac{1}{3}}}{\xi^2}sin^2\left(\frac{\vartheta_2}{2}\right) \quad (S55)$$

we obtain

$$f_3\left(\hat{G}^{SD}\right) = \frac{1}{64\,\pi^2}\,\hat{G}^{SD}\left(\frac{\hat{G}^{SD}}{\tau_{0D}}\right)^{2/3}\left(\frac{R_0^{DD}}{R}\right)^4 \iint_0^{up(\epsilon)} dx\,dy \iint_0^{2\pi} d\varphi_1 d\varphi_2 \left\{A(x,y,\varphi_1,\varphi_2,\epsilon) - \frac{\hat{G}^{SD} w_{x_2x_1}^{DD}}{1 + 2\,\hat{G}^{SD} w_{x_2x_1}^{DD}}\right\} \qquad \text{(S56)}$$

The upper limit of integration is equal to $up(\epsilon) = 4\left(\frac{R_0^{DD}}{R}\right)^{-2}\left(\frac{\hat{G}^{SD}}{\tau_{0D}}\right)^{-\frac{1}{3}}$.
In experiments with the standard range of parameter ξ and typical values of ϵ used in the Stehfest [24] procedure for inverting Laplace's transform, the integral in the expression for $f_3\left(\hat{G}^{SD}\right)$ in Eq. (S56) remains relatively constant and equals approximately -13.66269. Thus, the searched function is $f_3\left(\hat{G}^{SD}\right) \approx -0.02163\,\hat{G}^{SD}\left(\frac{\hat{G}^{SD}}{\tau_{0D}}\right)^{2/3}\left(\frac{R_0^{DD}}{R}\right)^4$

Finally,

$$\hat{\mathrm{A}}_3^{DDD}\left(\mathbf{0}, \hat{G}^{SD}\right) \approx -\,\hat{G}^{SD}\,(N-1)(N-2)\cdot 0.02163\left(\frac{R_0^{DD}}{R}\right)^4\left(\frac{\hat{G}^{SD}}{\tau_{0D}}\right)^{\frac{2}{3}} \qquad \text{(S57)}$$

The sum of all three-body multigraphs with loops $\hat{\mathrm{L}}_3^{DDD}\left(\boldsymbol{k}, \epsilon, \hat{G}^{SD}\right)$ defined in the Eq. (17) and (18) can be calculated exactly. Firstly, the function $B_2^{SD}(\epsilon)$ is calculated in a similar way as function $\hat{\Sigma}_2^{DD}$ in Supporting Information B, to obtain

$$\frac{B_2^{SD}(\epsilon)}{S} \approx -\frac{\hat{G}^{SD}}{36}\sqrt[3]{2}\,\sqrt{3}\,\pi\left(\frac{R_0^{DD}}{R}\right)^2\left(\frac{\hat{G}^{SD}}{\tau_{0D}}\right)^{\frac{1}{3}} \qquad \text{(S58)}$$

Next, the integral in Eq. (17) calculated in spherical coordinates on a sphere with a radius R and using the same expansions of the logarithm and arctangent functions in series as in the two-body approximation is

$$\frac{1}{S}\int d\boldsymbol{r}_{12}\frac{\hat{G}^{SD} w_{x_2x_1}^{DD}\left(1 + \hat{G}^{SD} w_{x_2x_1}^{DD}\right)}{\left(1 + 2\,\hat{G}^{SD} w_{x_2x_1}^{DD}\right)^2} \approx \frac{\sqrt{3}\,\sqrt[3]{2}\,\pi}{54}\left(\frac{R_0^{DD}}{R}\right)^2\left(\frac{\hat{G}^{SD}}{\tau_{0D}}\right)^{\frac{1}{3}} \qquad \text{(S59)}$$

Finally,

$$\hat{\mathrm{L}}_3^{DDD}\left(\boldsymbol{k}, \epsilon, \hat{G}^{SD}\right) \approx -(N-1)(N-2)\,\frac{\hat{G}^{SD}}{324}\sqrt[3]{4}\;\pi^2\left(\frac{R_0^{DD}}{R}\right)^4\left(\frac{\hat{G}^{SD}}{\tau_{0D}}\right)^{\frac{2}{3}} \qquad \text{(S60)}$$

The sum of all three-body multigraphs with nodes $\hat{\mathrm{N}}_3^{DDD}\left(\boldsymbol{k}, \epsilon, \hat{G}^{SD}\right)$ defined in Eq. (19) and (20) can also be calculated exactly. The function $\hat{\mathrm{N}}_3^{DDD}\left(\boldsymbol{k}, \epsilon, \hat{G}^{SD}\right)$ can be written in an equivalent form

$$\hat{\mathrm{N}}_3^{DDD}\left(\mathbf{0}, \epsilon, \hat{G}^{SD}(\epsilon)\right) = (N-1)^2\left(\hat{G}^{SD}\right)^3\left(\frac{1}{S}\int d\boldsymbol{r}_{13}\frac{w_{x_3x_1}^{DD}}{1 + 2\,\hat{G}^{SD} w_{x_3x_1}^{DD}}\right)^2 \qquad \text{(S61)}$$

The integral in brackets has already been computed before, see Eq. (S45) and (S49), so we get

$$\hat{\mathrm{N}}_3^{DDD}\left(\mathbf{0}, \epsilon, \hat{G}^{SD}(\epsilon)\right) \approx (N-1)^2\,\frac{\sqrt[3]{4}\;\pi^2}{432}\hat{G}^{SD}\left(\frac{R_0^{DD}}{R}\right)^4\left(\frac{\hat{G}^{SD}}{\tau_{0D}}\right)^{\frac{2}{3}} \qquad \text{(S62)}$$

Supporting Information C: Monte Carlo simulations

In our study, we employed the Monte-Carlo simulation technique to investigate the time dependence of a model characterized by stochastic growth or change. This technique relies on a sequence of random numbers generated during the simulation to guide the occurrence and type of specific processes within the simulated system. Specifically, we utilized a ”step by step” Monte-Carlo method based on the Gillespie procedure [25] , which has been adapted and applied to describe energy transfer and migration in various systems, including disordered concentrated two-component systems, [26–28] polypeptides labeled with multiple fluorophores, [29] porous nanolayers, [30] uniaxial oriented polymer films, [31] and core-shell spherical nanoparticles. [21] The block diagram of the Monte-Carlo simulation, corresponding to a single simulation run, is illustrated in Fig. S12. To obtain reliable statistics of the phenomena, a sufficiently large number of steps corresponding to specific processes should be performed during the simulation. The convergence and stability of the results can be assessed by tracking how subsequent runs affect the average outcome. If the number of runs is too small, subsequent runs will introduce significant variations in the average value. Conversely, if the results converge to the desired outcome, the variation of the average becomes negligibly small, in accordance with the Central Limit Theorem. In our simulations, the results were averaged over 10^7 runs, which ensured stable and accurate results with minimal error.

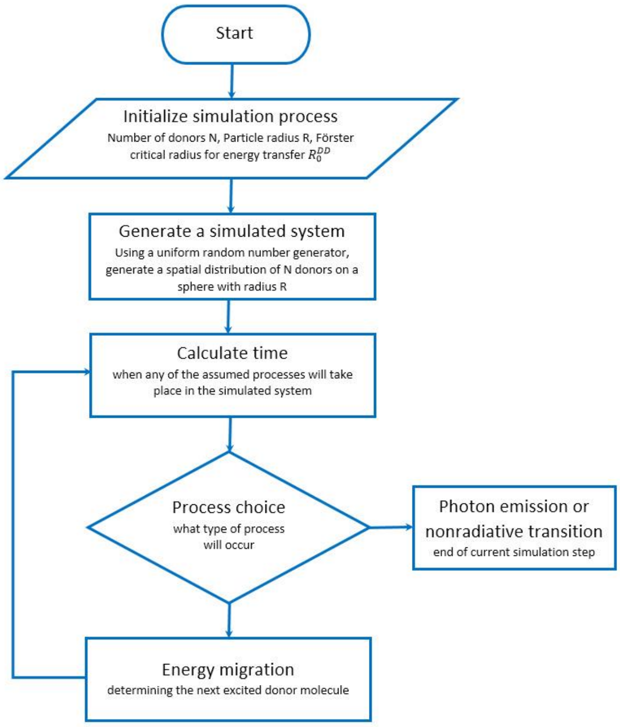

Figure S12: Block diagram of one step in the Monte Carlo simulation.

The Monte-Carlo method provides high-accuracy predictions not only for observables such as emission anisotropy decay but also for valuable insights into the mechanism and locality/nonlocality of energy migration. This includes characteristics that are not directly measurable, such as the mean displacement distance of excitation energy presented in this work. Furthermore, this simulation approach offers insights into other non-measurable characteristics, such as the mean number of excitation energy jumps among molecules, the mean visiting time of excitation in specific molecular ensembles, quantum efficiency, and emission anisotropy of molecules involved in energy migration at different excitation levels (primary, secondary, tertiary, etc.). Overall, the Monte-Carlo method proves to be a powerful tool for accurately simulating fluorescence properties and investigating energy

migration dynamics in complex systems. It benefits both experimental and theoretical research endeavors by providing valuable insights and aiding in the understanding of complex phenomena.
